# DSIR: Assessing the Design of Highly Potent siRNA by Testing a Set of Cancer-Relevant Target Genes

**DOI:** 10.1371/journal.pone.0048057

**Published:** 2012-10-30

**Authors:** Odile Filhol, Delphine Ciais, Christian Lajaunie, Peggy Charbonnier, Nicolas Foveau, Jean-Philippe Vert, Yves Vandenbrouck

**Affiliations:** 1 CEA, DSV, iRTSV, Laboratoire de Biologie du Cancer et de l’Infection, Grenoble, France; 2 INSERM U1036, Grenoble, France; 3 Université Grenoble I, Grenoble, France; 4 Mines ParisTech, Centre for Computational Biology, Fontainebleau, France; 5 Institut Curie, Paris, France; 6 INSERM U900, Paris, France; 7 CEA, DSV, iRTSV, Laboratoire de Biologie à Grande Echelle, Grenoble, France; 8 INSERM U1038, Grenoble, France; Niels Bohr Institute, Denmark

## Abstract

Chemically synthesized small interfering RNA (siRNA) is a widespread molecular tool used to knock down genes in mammalian cells. However, designing potent siRNA remains challenging. Among tools predicting siRNA efficacy, very few have been validated on endogenous targets in realistic experimental conditions. We previously described a tool to assist efficient siRNA design (DSIR, Designer of siRNA), which focuses on intrinsic features of the siRNA sequence. Here, we evaluated DSIR’s performance by systematically investigating the potency of the siRNA it designs to target ten cancer-related genes. mRNA knockdown was measured by quantitative RT-PCR in cell-based assays, revealing that over 60% of siRNA sequences designed by DSIR silenced their target genes by at least 70%. Silencing efficacy was sustained even when low siRNA concentrations were used. This systematic analysis revealed in particular that, for a subset of genes, the efficiency of siRNA constructs significantly increases when the sequence is located closer to the 5′-end of the target gene coding sequence, suggesting the distance to the 5′-end as a new feature for siRNA potency prediction. A new version of DSIR incorporating these new findings, as well as the list of validated siRNA against the tested cancer genes, has been made available on the web (http://biodev.extra.cea.fr/DSIR).

## Introduction

RNA interference (RNAi) is the process through which a double-stranded RNA (dsRNA) silences gene expression, either by inducing degradation of sequence-specific complementary messenger RNA (mRNA) or by repressing translation [1]. The endogenous mammalian RNAi pathway uses noncoding microRNAs (miRNAs) to modulate gene expression through translational repression and/or mRNA cleavage, by targeting the 3′ untranslated regions (3′UTRs) of mRNA with which they share partial complementarity [2]. Modeled on these miRNAs, chemically synthesized dsRNA reagents shorter than 30 nucleotides were found to trigger a sequence-specific RNAi response without inducing the cell’s innate immune defenses in mammalian systems [3], [4]. Duplex small interfering RNA molecules (siRNA) theoretically have the potential to specifically inhibit the expression of almost any target gene. Therefore, they have become a widespread molecular tool representing a powerful means to study gene function [5], [6]. Preclinical studies and some early clinical trials have already demonstrated that siRNAs have potential as novel therapies for a wide range of diseases, including cancer [7].

For RNAi to be reliable, siRNAs must be designed with care, to ensure the efficacy and the specificity of the selected sequence for its target gene [6], [8]. siRNA efficacy is a measure of the cooperative partnership between the guide-strand and the RISC machinery leading to mRNA cleavage. In contrast, siRNA specificity corresponds to accurate recognition of target sites, avoiding unwanted side-effects (e.g., “off-target” effect). Studies based on both experimental data and computational approaches have reported that the secondary structure of targets and their accessibility were also important, although less so, in determining siRNA activity [9], [10], [11], [12].

Several programs and web servers have been developed to automate siRNA design. These implement design rules based on nucleotide preferences at specific positions, sequence features, potential hairpin formation, stability profiles, energy features, weighted patterns and secondary structure of the target mRNA. These siRNA features have been summarized in review articles [see [13], [14]]. Optimal siRNA features are best determined based on experimental data. Huesken et al. [15] published a set of 2182 randomly selected siRNA, which were assayed using a high-throughput fluorescent reporter gene system. This led to the development of a new generation of algorithm based on machine learning techniques which has significantly improved siRNA design, with a reported Pearson correlation coefficient of 0.66–0.67 between measured and predicted efficacy. Recently, an evaluation of various siRNA-designing tools concluded that *Biopredsi* [15], *Thermocomposition* [16] and *DSIR,* a computational model developed by us, [17] were highly accurate and reliable predictors of active siRNA [18], [19]. DSIR is based on a linear model combining particular nucleotides at given positions and specific motifs on the siRNA guide-strand, including 2-nt overhangs at the 3′ end [17]. This combination provides efficient siRNA, probably due to high rates of RISC binding and/or Ago2-mediated cleavage of the target mRNA complementary strand [18].

However, features that are crucial for the optimal prediction of efficient siRNA are still debated [13], [14], [20]. Therefore, despite these improvements, it remains to be determined which algorithm and web tool is optimal, and how well predicted siRNAs behave in realistic experimental conditions. On the one hand, none of currently available siRNA design methods covers the full siRNA-machinery process. Thus, although the relative contribution of features such as siRNA efficiency, specificity or target accessibility have been scrutinized independently for their role in the resulting final knockdown, a single global study has yet to be performed. On the other hand, very heterogeneous datasets have been used to design most of these computational models predicting siRNA efficacy [21]. These datasets were obtained using different methods to measure mRNA knockdown, different siRNA concentrations and lengths (from 19 to 21 nt), and used various cell types or transfection systems. All these differences are summarized in Table 1. Notably, the Huesken dataset, frequently used to develop siRNA design algorithms [15], [16], [17], [22], was produced using a plasmid coding for both an exogenous reporter gene bearing target cDNA inserts with its 3′ untranslated region (UTR), and a reference gene [15]. This measurement system may not be appropriate when investigating how an siRNA behaves towards its mRNA target in its natural cellular environment. Another problem encountered when combining data from heterogeneous sources is the lack of detailed information regarding the target sequence [23].

**Table 1:** Experimental siRNA datasets used to establish computational models predicting siRNA efficacy.

Experimentaldataset name	Total numberof siRNA tested	Total number oftarget genes	Knockdownmeasurementmethods	Cell type	siRNA finalconcentration(nM)	Design algorithmtrained withthis dataset
ISIS	80	8	qRT-PCR	T24	100	Vickers et al., 2003 [53]
Dharmacon	180	2 (Firefly luciferase, Human cyclophilin B)	Reporter gene plasmid	HEK293	100	Reynolds et al., 2004 [54]Saetrom & Snöve, 2004 [55]
Novartis (also referredas Huesken dataset)	2182	34	Reporter system with fusion transcripts	NCI-H1299, HeLa	50	Huesken et al., 2005 [15]Shabalina et al., 2006 [16]Vert et al., 2006 [17]
Sloan-Kettering	601	4	Reporter gene systemImmunofluroescenceFunctional assay	HeLa, HUVEC	100	Jagla et al., 2005 [56]

To evaluate the accuracy of automated siRNA design tools in a realistic experimental environment, we focused on the DSIR design tool and systematically investigated how well it behaves in “real-life” by measuring mRNA knockdown in a standardized cell-based assay. To do this, we established a controlled, normalized experimental procedure for siRNA transfection and quantitative real-time time PCR (qRT-PCR) measurements. We assessed the silencing potency of a set of DSIR-designed, 21-nt siRNA duplex sequences directed against ten human cancer-related target genes. Using this approach, we quantified the overall predictive power of the siRNA design algorithm DSIR. It also allowed us to further investigate factors potentially improving DSIR performance.

## Materials and Methods

### 1 Target and siRNA Selection

We initially selected eight human genes known to be key molecular components in cell transformation and cancer processes (Table 2). These genes were retained for therapeutic evaluation in preclinical studies related to various tumor models by the “siRNA consortium”, a project funded by the Ligue Nationale Contre le Cancer. siRNA targeted against regions of the full length target mRNA sequence were designed with DSIR. The 21-nt model included 2-nt 3′ overhangs [17]. siRNAs with more than 80% predicted extinction activity were retained, without discarding siRNA containing polynucleotide tracts. Among these siRNA sequences, siRNA that overlapped or were too closes spaced on target sites were not retained if possible. The final set consisted of 88 siRNA duplexes. A positive control siRNA targeting CSNK2B (previously validated by [24]), and a negative control siRNA against GFP were also included in this first list of siRNA.

**Table 2:** mRNA target description, features and number of siRNA tested in the overall study.

Target official symbol*	RefSeq accession number	mRNA definition (according to NCBI RefSeq division)	Totallength(nt)	CDSlength	Numberof exons	Numberof siRNA tested
BCL2L1	NM_138578	Homo sapiens BCL2-like 1, nuclear gene encoding mitochondrialprotein, transcript variant 1 (Bcl_XL)	2575	702	3	12
CSNK2A1	NM_001895	Homo sapiens casein kinase 2, alpha 1 polypeptide (CK2α)	2732	1176	13	10
CSNK2A2	NM_001896	Homo sapiens casein kinase 2, alpha prime polypeptide (CK2α’)	1674	1053	12	10
CSNK2B	NM_001320	Homo sapiens casein kinase 2, beta polypeptide (CK2β)	1128	648	7	10
ERCC1	NM_001983	Homo sapiens excision repair cross-complementing rodent repairdeficiency, complementation group 1, transcript variant 2	3400	894	10	9
ERCC2	NM_000400	Homo sapiens excision repair cross-complementing rodent repairdeficiency, complementation group 2, transcript variant 1	2568	2283	23	27
HIF1A	NM_001530	Homo sapiens hypoxia-inducible factor 1, alpha subunit (basichelix-loop-helix transcription factor), transcript variant 1 (HIF1α)	4082	2481	15	10
HDAC6	NM_006044	Homo sapiens histone deacetylase 6	4099	3648	29	25
LIG1	NM_000234	Homo sapiens ligase I, DNA, ATP-dependent (LIG1)	3083	2760	28	8
PARP1	NM_001618	Homo sapiens poly (ADP-ribose) polymerase 1 (PARP1)	4001	3045	23	8

* : according to HGNC.

In a second round of experiments, an additional set of 40 siRNA directed against two of the eight genes targeted in the first round, ERCC2 and HDAC6, and two novel target genes, PARP1 and DNA Ligase 1 (LIG1) was prepared. This set was used to check the contribution of intrinsic target features identified during the first round of experiments. The criteria for siRNA inclusion in the second round were: >80% extinction activity predicted by DSIR, a location in the coding sequence (CDS) part of the target, no polynucleotide tracts and no potential off-targets allowed, an extended panel of target sites by complementary coverage of the overall transcript sequence with respect to the siRNA sequence designed for the first set. Details of the two sets of siRNA are listed in supplementary Table 1.

#### siRNA synthesis

All siRNA for the knockdown of the ten human genes were synthesized as duplexes by SIGMA Proligo as 21 mers (Supplementary Table S2).

### 2 Cell Culture and Transfection

HeLa cells were cultured in Dulbecco’s modified Eagle’s medium (DMEM) containing 10% fetal bovine serum (FBS), 100 unit/ml penicillin, 100 mg/ml streptomycin, in a humidified incubator at 37°C in 5% CO_2_. About 1×10^5^ cells were inoculated in 12-well plates and cultivated for 24 h. Medium was changed to 0.4 ml of OPTI-MEM (Gibco) before transfecting cells with 20 nM siRNA using Oligofectamine (Invitrogen) according to the manufacturer’s instructions. Transfected cells were incubated for 5 h. Cells were then washed once with PBS, DMEM 10% FBS was added, and cells were cultured for a further 72 h (except for BCL2L1 where cells were cultured for only 24 h).

### 3 Real-time PCR Assay and Measurements

Three days after siRNA transfection, cells were harvested and RNA was isolated using Absolutely RNA miniprep kit (Stratagene). RNA concentration was determined using a NanoDrop. Reverse Transcription was performed with the StrataScript QPCR cDNA Synthesis kit (Stratagene), according to the manufacturer’s instructions. Gene-specific primers (forward and reverse) were designed to be compatible with a single qRT-PCR thermal profile such that multiple transcripts could be analyzed simultaneously. PrimerQuest (http://www.idtdna.com/SCITOOLS/Applications/PrimerQuest/) was used for their design. Primer sequences are listed in Supplementary Table S2. Quantitative PCR was performed with FullVelocity SYBR Green QPCR Master Mix using the primer concentrations indicated in Table 2. PCR conditions (primer concentrations, cDNA quantity) were optimized and PCR efficiency was determined for each target gene. PCR reaction mixtures (25 µl) were placed in the Mx3000P instrument where they underwent the following cycling program, optimized for a 96-well block: 95°C for 5 min, immediately followed by 45 cycles of 10 sec at 95°C and 30 sec at 60°C. At the end, PCR products were dissociated by incubating for 1 min at 95°C and then 30 sec at 55°C, followed by a ramp up to 95°C. PCR quality and specificity were verified by analyzing the dissociation curve. For each set of primers, a no-template control (NTC) and a no-reverse-amplification control (NAC) were included. qRT-PCR reactions were run in triplicate, and quantification was performed using the comparative threshold-cycle method. Quantitative PCR data were comparatively analyzed using MxPro software (Stratagene, “Comparative Quantification” application) with either the 36B4 or hHPRT amplification signal as internal “normalizer” (to correct for total RNA content) and labeling mock tranfected sample as “calibrator”. Results are expressed as the relative change in expression compared to the control. Experiments were performed in triplicate for each sample.

### 4 Quantitative Real-time PCR Data Normalization and Statistical Analysis

Each PCR run included three biological replicates for each treatment analyzed. In addition, all experiments were repeated three times independently, with different biological samples. The whole procedure was also performed twice, using two different normalizing genes. 18 extinction ratio estimates *Q* ^*^ were obtained from this data, based on the following standard formula:
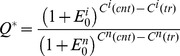
where 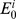 is the amplification rate at the early stage (ARES) of the process for the target molecule, 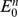 is the ARES for the normalizing molecule, and *C* is the number of cycles required to reach a pre-defined level of signal. It would have been convenient to combine independence and homogeneity assumptions, to determine confidence regions. However, here this process is clearly questionable, since some pairs of experiments share design conditions that others do not (for instance the same run, or the same normalizing gene). For this reason, a different analysis was performed, the details of which can be found in the supplementary material. Extinction values for each siRNA molecule are provided in Supplementary Table S3.

### 5 Western Blot Assay

Three days after siRNA transfection, HeLa cells were harvested for protein extraction in RIPA buffer (Tris HCl pH 7.4 10 mM, NaCl 150 mM, SDS 0.1%, Na Deoxycholate 0.5%, EDTA 1 mM, Triton X100 1%, Leupeptin 5 µg/ml, Aprotinin 5 µg/ml). Proteins were separated by electrophoresis on a 12% SDS-PAGE gel before transfer to PVDF membrane for 1 h at 100 V. Primary antibodies: polyclonal CK2alpha antibody (αCoc) [25] and monoclonal Hsp90 antibody (clone 16F1 from Stressgen) were diluted 1/500 in PBS containing 3% non-fat milk powder. Goat anti-rabbit or anti-mouse IgG -peroxidase (Interchim) were used as secondary antibodies, diluted 1∶5000 in PBS. Secondary antibody binding was revealed using ECL plus reagents (Amersham, #RPN 2105).

### 6 Statistical Analyses

The influence of various factors on the siRNA efficacy measured was analyzed by fitting linear models and testing the significance of each term using R statistical analysis software (http://www.r-project.org/). The significance of each model term and of additional explanatory variables was tested using ANOVA statistics.

### 7 Secondary Structure of Target mRNA and Accessibility

Target site accessibility was systematically evaluated either using the SFold web server (http://sfold.wadsworth.org), where the probability profile of predicted target accessibility can be visually inspected using the siRNA module (see supplementary Figure S2). Alternatively, the RNAplfold program (Vienna package release 1.7.2) was used with the previously defined optimal folding parameters (W = 80, L = 40 and u = 16), as described in [22]. The higher the RNAplfold probability, the more accessible the target site is. The RNAplfold probabilities for each siRNA sequence are listed in Supplementary Table S3.

### 8 Potential Off-target Search

Potential off-target gene knockdown was detected by applying an in-house implementation of the Wu-Manber algorithm, a variant of the Baeza-Yates shift-add method [26]. In contrast with Blast, this program performs an exact search of a given pattern (the short siRNA sequence) in a sequence databank (NCBI RefSeq division, release 32) allowing for mismatches. In our study, the default value for the number of mismatches allowed was set to three, as recommended [13]. Identity between mRNA and siRNA seed-regions encompassing nucleotides 2–8 of the antisense strand were computed. This involved running the Wu-Manber implementation with no mismatch allowed, and checking heptamer seed sequences against a 3′UTR mRNA sequence databank (built from the human genes listed in RefSeq, release 48). The number of 3′UTR sequences matched in the global transcriptome, the number of seeds matching a 3′UTR sequence one, two and three or more times are all reported (supplementary Table S3). These features have been suggested to be linked to RNAi off-targets [27], [28].

## Results

### 1 Experimental Setup

**Figure 1:**
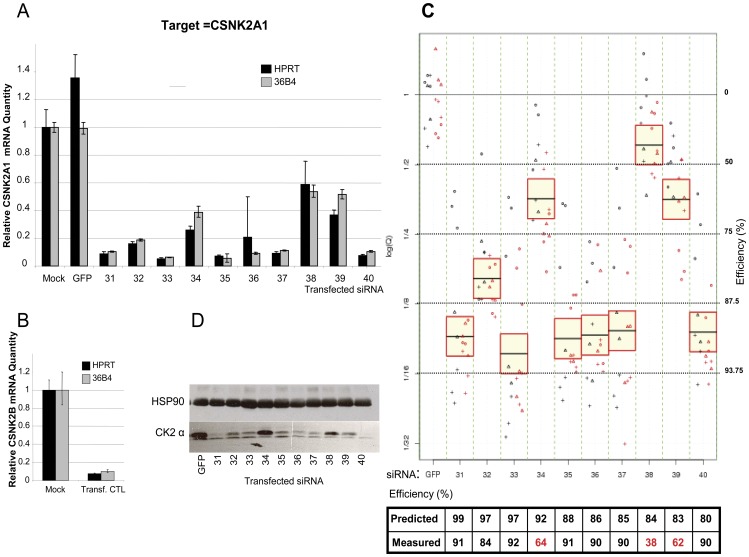
Complete molecular analysis of target gene silencing: CSNK2A. HeLa cells were transfected with ten siRNA targeting CSNK2A, and two control siRNA, (GFP as a negative control and CSNK2B as a positive control) at a final concentration of 20 nM using Oligofectamine reagent. An additional control consisted in mock transfection of cells (without siRNA). Three days later, RNA and proteins were extracted for further analysis. **A.** Effect of siRNA treatment from a typical experiment. For each siRNA the relative quantity of the target mRNA to HPRT (black) or 36B4 (grey) was plotted using the comparative analysis module in MxPro software (Stratagene). **B.** Transfection efficiency control. For each experiment, transfection efficiencies were checked by quantifying gene silencing relative to a control siRNA of known efficiency. Results of experiments where this control did not silence expression by more than 70% were excluded from the dataset because transfection efficiency was considered to be poor. **C.** Box plot representation of siRNA efficiency for 10 sequences. For each siRNA, efficiency predicted by DSIR and measured efficiency are indicated. Measured efficiency was statistically determined from triplicate RT-qPCR quantification of target mRNA after siRNA treatment, based on three independent experiments. Expression levels were normalized to HPRT (black) and 36B4 (red) house-keeping genes. Log(Q) = 1 represents no reduction in target mRNA after treatment and log(Q) = 1/4 equates to approximately 75% efficiency. See section 2.6 for further details of the statistical analysis. Overall siRNA efficiency and significance values are provided in supplementary material. **D.** Western blot analysis of silencing efficiency, using anti-CK2alpha antibody. Protein loading was normalized for Hsp90 levels.

The main goal of the present study was to assess the efficacy of siRNA sequences designed by DSIR. Efficacy was assessed endogenously by measuring the percentage of remaining non-cleaved mRNA relative to a control, non-targeted mRNA. A standardized procedure was established to avoid biases due to experimental conditions (i.e., cell type, transfection conditions, concentration, quantitative real-time RT-PCR, etc). In a first round of experiments, we focused on eight target genes related to cancer. ERCC1 and ERCC2 are known to be involved in the nucleotide excision repair (NER) pathway and are essential to the repair of cisplatin-induced DNA adducts (excision repair cross-complementing rodent repair deficiency) [29]. HDAC6 displays histone deacetylase activity and represses transcription [30]. BCL2L1 (Bcl-X_L_) is an antiapoptotic Bcl-2 family member and key regulator in the apoptotic process [31]. HIF1A (HIF1α) is a transcription factor induced by hypoxia [32]. To these, we added the three subunits of protein kinase CK2, (i) CSNK2A1 (CK2α), (ii) CSNK2A2 (CK2α’) and (iii) CSNK2B (CK2β) [33]. These mRNA target genes are described, their features, and the corresponding number of siRNA reagents tested are listed in Table 2. All these genes are naturally expressed in human HeLa cells, which are known to be easily transfectable. These cells were chosen to avoid any additional variation due to the transfection step. For each target, 10 or more 21-nt siRNA were designed with DSIR, and selected as described in Materials and Methods. Given that a high siRNA concentration may lead to off-target effects, and a low concentration can result in undetectable gene silencing, we aimed for a compromise by fixing the siRNA concentration at 20 nM. Fold-changes in gene expression were determined by the ΔΔCt method, with normalization to either 36B4 or HPRT house-keeping genes. This normalization strategy, based on two reference genes, has been demonstrated to be more reliable than normalization with a single reference gene [34]. HeLa cell transfection efficiency was checked in all the experiments by quantifying the effect of a previously validated siRNA [24] targeting CSNK2B. The experiment was considered as validated when this positive control siRNA down-regulated CSNK2B by at least 80%. We also developed a new model that allows for normalization with the two housekeeping genes, 36B4 and HPRT, together. Our analytical approach involved normalization using multiple reference genes and inter-run calibration. This helped to avoid error propagation and deviation between plates (see details in supplementary Material). Finally, since the effect of the siRNA is ultimately required at the product level, a western blot assay for CSNK2A1 protein was used to validate our observations. All of these results and their complete analysis are presented Figure 1 for this gene (and in supplementary Figure S1 for other target genes).

### 2 Assessing DSIR Design by Measuring siRNA Activity

The global siRNA efficiency rate per target gene is summarized in Table 3. To evaluate how successfully the DSIR design model designs siRNAs, we developed a grading system. siRNAs that yielded at least 70% target gene knockdown were considered highly efficient; other siRNA were considered either moderately efficient (from 50 to 70%) or inefficient (<50%). According to this ranking, the whole siRNA dataset contains more than 56% highly efficient, and 25% moderately efficient siRNA. This overall success rate shows that DSIR performs well in real experiments.

**Table 3:** Global silencing efficiency for each target gene.

Target name	ERCC1	CSNK2B	CSNK2A1	CSNK2A2	BCL2L1	HIF1A	ERCC2	HDAC6	ERCC2*	HDAC6*	PAR1*	LIG1*
siRNA efficiencyratio	8/9	7/10	7/10	10/10	7/12	5/9	4/15	3/13				
Ratio after filtering	8/9	7/8	7/10	10/10	7/10	5/9	4/13	3/12	5/12	5/12	8/8	7/8

siRNA efficiency ratio represents the number of siRNA with >70% silencing activity over the total number of siRNA tested experimentally. The ratio after filtering corresponds to the siRNA sequences remaining after removal of siRNA sequences targeting 5′ UTR regions. (*: Genes targeted in a second round test).

We next focused on each target separately to determine the success rate for extinction by counting the number of efficient siRNA sequences over the total number of siRNA tested for each gene (Table 3). This data reveals a striking heterogeneity of success rate from one target gene to the next, suggesting different reactions to the siRNA-mediated silencing pathway. Indeed, qRT-PCR measurements of the following transcripts, ERCC1, CSNK2B, CSNK2A1, CSNK2A2, reveal a satisfactory silencing profile (with 9/10, 7/10, 7/10 and 10/10 efficient siRNA, respectively). In contrast, ERCC2 and HDAC6 (4/15 and 3/13, respectively) seem to be quite resistant to silencing, with only a few efficient siRNA despite a higher number of siRNA reagents tested. Intermediate success is observed for HIF1A and BCL2L1, with efficient siRNA activity in 5/9 and 7/12 cases, respectively. This contrasted picture highlights that, in terms of silencing, all genes are not equal. This was somewhat surprising as expression levels for all these target genes was documented to be similarly low. Hence, if we discard target genes which are the most refractory to silencing, ERCC2 and HDAC6, DSIR predicts more than 70% highly efficient siRNA sequences. These observations highlight the non-negligible role played by the target mRNA when evaluating computational models predicting siRNA efficacy.

### 3 Relationship between siRNA Efficacy and Potential off-target Effects

**Figure 2:**
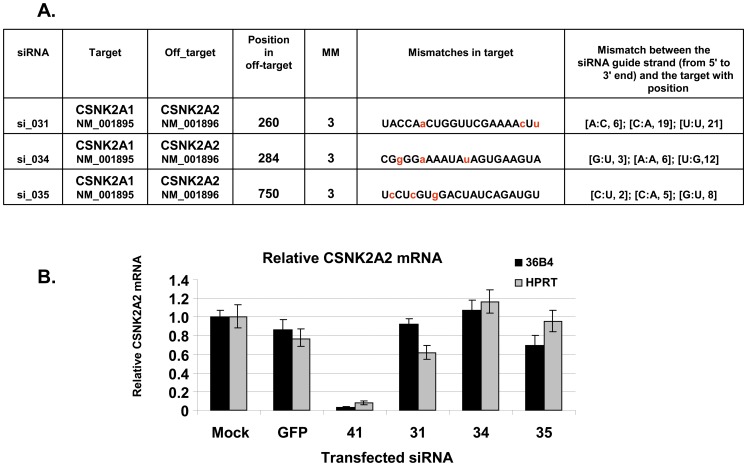
Mismatch effect on RNAi silencing activity. A. Three siRNA sequences targeted against CSNK2A1 were identified as potential off-targets for CSNK2A2, with three mismatches at different positions (in lowercase). HeLa cells were transfected with three siRNA against CSNK2A1 (CK2α) (si_31, si_34 and si_35), or against CSNK2A2 (CK2α’) (si_41) and GFP control siRNA. All siRNAs were used at a final concentration of 20 nM, and transfected using Oligofectamine reagent. Mock transfected cells (without siRNA) were also included. Three days later, RNA was extracted for further analysis. B. Relative CSNK2A2 mRNA quantity determined by qRT-PCR. For each siRNA, the quantity of target (or off-target) mRNA relative to HPRT (black) or 36B4 (grey) was plotted.

It has been demonstrated that siRNA may non-specifically target unrelated genes presenting only partial sequence complementarity. This is known as off-target effects (OTE). Two types of OTE are now distinguished [35]. The first of these is OTE mediated by Ago2 acting on targets which are highly similar to the actual siRNA targets. This appears to be very potent, although this type of off-target effect is relatively rarely encountered. The second type of OTE has been observed on targets that contain seed matches in their 3′UTR region [36]. This involves a mechanism that appears to be similar to that of miRNA regulation [27], [36], [37]. Since OTE could account for lower efficiency by a dilution effect, we investigated this aspect by systematically screening each siRNA for potential off-targets sites. First, we observed that neither the number of partial complementary off-targets, nor the determinants associated with the seed complement frequency class were significantly related to siRNA efficiency (see below 3.4). In addition, Du et al. [38] reported that the position of the mismatch within the duplex and the identity of the base constituting the mismatch could influence siRNA functionality. To study the effects of mismatches between siRNA and mRNA target more precisely in relation to RNAi silencing activity, we focused on the cross-reaction that could exist between the CSNK2A2 gene target and siRNA directed against CSNK2A1, for which three siRNA sequences from our dataset (si_031, si_034 and si_035) have been identified as having potential OTE (see Figure 2A). Although these three siRNA have been shown to drastically reduce CSNK2A1 expression, none of them had any effect on the level of CSNK2A2 transcript, as revealed by qRT-PCR (see Figure 2B). Furthermore, we noticed that the position of the mismatches for each siRNA guide-strand sequence (si_031: 6,19,21; si_034: 3,6,12; si_035: 2,5,8) within the CSNK2A2 transcript were not necessarily in agreement with the tolerated position-dependent mismatches described previously [38]. For example, position 12 was found to be weakly tolerated, whereas positions 1, 2, 5, 7, 8, 18 and 19 were found to be well-tolerated. In our case, the active siRNA (si_035) contains three high-tolerance positions which could also have caused CSNK2A2 knockdown. However, no effect was observed. This suggests that this combination is not tolerated, or that OTE due to near-perfect complementarity is much more complex.

**Figure 3:**
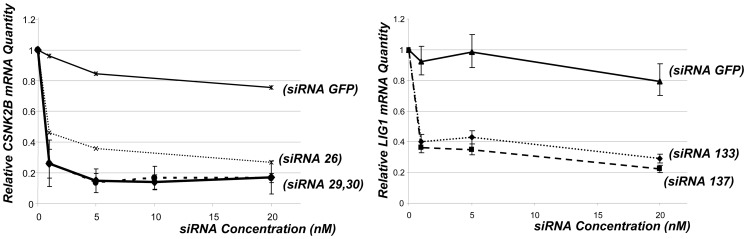
Extent of siRNA silencing does not correlate with transfected siRNA concentration. A range of concentrations of three siRNAs directed against CSNK2B, all with high global efficiency (siRNA_26, _29 and si_30) (panel A), and two siRNA directed against LIG1 (siRNA_133 and _137) (panel B) were transfected into HeLa cells to determine the relationship between overall efficiency and amount transfected. siRNA_GFP was used as a negative control. The graph shows mean values +/− standard deviations from RT-qPCR quantification of two independent experiments normalized to the HPRT house-keeping gene.

Another way to minimize OTE is to lower the siRNA concentration. Our validation was performed with 20 nM siRNA, but we also tested lower quantities to further assess efficient sequences. As shown in Figure 3, concentrations as low as 1 nM are as efficient as 20 nM for three siRNA sequences directed against CSNK2B and two siRNA sequences directed against LIG1 (gene target added in the second round of tests, see below). These results indicate that siRNA designed by DSIR may be powerful even in “physiological” conditions, where concentrations can be as low as 1 nM.

### 4 Exploring the Features Affecting siRNA Potency

All the siRNA designed using DSIR had a predicted potency of at least 80%. The contribution and significance of several previously published siRNA design criteria or factors that are thought to play a role in gene silencing, and which are not explicitly taken into account by the DSIR model, were analyzed. The influence of some of these features on the variations observed in the efficacies of the 88 siRNA tested in the first round of experiments was analyzed. To do so, we first fitted a linear model to explain the siRNA efficacies measured as a function of 13 features: (1) the DSIR score, (2) the target gene, (3) the position in the target gene (in bp relative to the 5′end), (4) the location in the target gene (5′ UTR, CDS or 3′ UTR), (5) the number of potential off-targets for the siRNA, (6) the absence or presence of a polynucleotide tract (with > = 4 identical consecutive nucleotides) in the siRNA, (7) the number of hits for the siRNA on the global human transcriptome, (8–10) the number of mRNA sequences matching in their 3′UTR region, (11) the length of the target exon, (12) the presence of an exon-exon junction at the target site, and (13) target site accessibility, computed as described in Materials and Methods. All of this information is provided in supplementary Table S3. ANOVA tests with a significance threshold of 5% for the P-values revealed that only three of these thirteen covariates correlated significantly with efficacy; these were as follows: the identity of the target gene, the position in the target gene, and the location in the target gene (as defined above). In particular, in the narrow range above 80%, the DSIR score does not seem to be significantly correlated to the efficacy measured, suggesting that it should be used only to select siRNA above a threshold, but not to rank them. Moreover, none of the other covariates that have been suggested as design criteria (number of off-targets, presence of a polynucleotide tract, target site accessibility) showed any evidence of being able to predict the efficacy measured in our set of siRNA (not shown).

**Figure 4:**
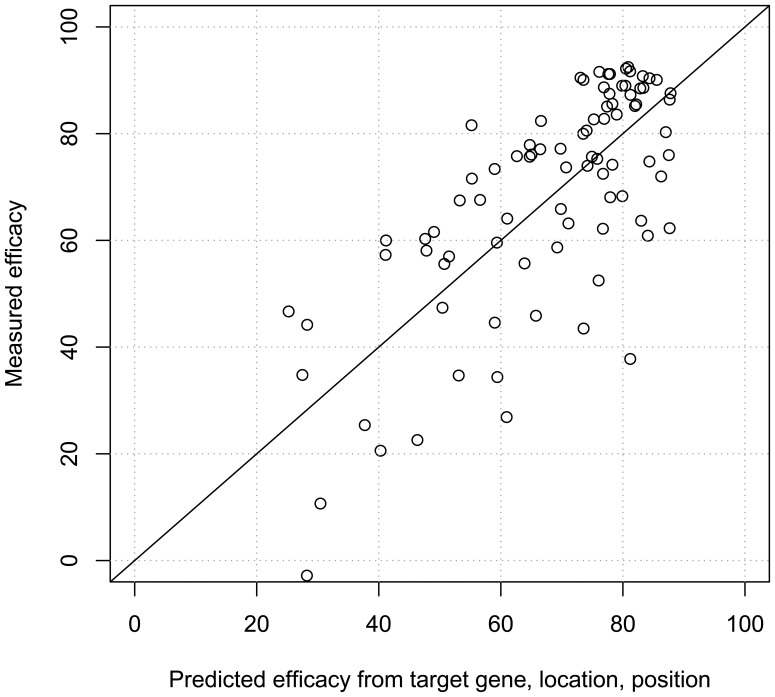
Influence of target gene identity, location and position in the target mRNA. Measured vs. fitted efficacy calculated using a linear model was plotted. The linear model includes three factors as covariates: the target gene, the target site location (5′UTR, CDS or 3′UTR), and the target site position (number of bp from the 5′ end). All three covariates are statistically significant in this linear model.

We therefore re-estimated a linear model with only the three significantly contributing covariates. First, the location of the target site in the 5′ UTR, the CDS or the 3′ UTR has a significant impact on the efficacy measured (Pval <0.0003). On average, siRNAs targeting the CDS or the 3′UTR are more effective (roughly 39% and 13%, respectively) than those targeting the 5′UTR. Second, in addition to straightforward UTR/CDS location, we observed a significant negative contribution for siRNA target sites positioned at greater distances (measured in base pairs) from the 5′ end of the target mRNA (Pval <0.0002). siRNA potency decreases by roughly 1% per 100 bp, on average, as the target site moves away from the start codon of the target mRNA (first ATG after the 5′UTR). Third, and more significantly, the model confirms that besides positional factors, the targeted gene itself contributes considerably to explaining the differences in siRNA efficacies (Pval <0.00002). For example, taking BCL2L1 as mean reference, siRNAs targeting HDAC6 and ERCC2 have significantly decreased efficacy, with an average of 22% and 11%, respectively, while siRNA targeting ERCC1, CSNK2A1, CSNK2A2 and CSNK2B are significantly more potent by 7%, 9%, 10% and 8%, respectively. As shown in Figure 4, the resulting linear model with three covariates precisely explains the differences in efficacy observed for the 88 siRNA, all of which share a high predicted potency by DSIR.

### 5 Observation and Validation of the Position Effect on siRNA Potency

To our knowledge, of the three features that were found to be significantly predictive for siRNA efficacy (target gene, position and location of the targeting site), position has not been mentioned previously. Although significant when all 88 siRNA were pooled together in the statistical analysis, we wanted to check whether this effect is also present and detectable in all target genes. Focusing only on siRNA targeting the CDS, we therefore checked, within each target gene, whether position is a significant predictor for siRNA potency. For HDAC6, a very strong negative positional effect (P<0.0005) was observed. In contrast, the negative effect appears to be present but not significant for ERCC1 and ERCC2 (P<0.2), and, remarkably, no positional effect is detectable for the other genes tested. Although the power of statistical tests is limited when considering each target gene in turn, this suggests that the positional effect on siRNA efficacy, while significant on average, is only found for some target genes.

To further evaluate this positional effect, we designed and tested a second series of 40 siRNA targeting two genes already present in the first series (ERCC2 and HDAC6), and two new genes (PARP1 and LIG1). LIG1 codes for DNA ligase I, with functions in DNA replication and in the base excision repair process, while PARP1 codes for a chromatin-associated enzyme, poly(ADP-ribosyl)transferase, which modifies various nuclear proteins by poly(ADP-ribosyl)ation [39]. This series of tests confirmed the very clear positional effect on HDAC6 (P<0.003). However, although slightly negative correlations were observed between the sites targeted by the siRNA and their efficacy knocking down LIG1 and PARP1, the position effect is not statistically significant. To summarize, the novel effect of targeting site on siRNA efficacy was identified overall, and validated for a group of genes. When taken individually, this effect does not seem to be present on all genes.

### 6 DSIR, a Website Dedicated to the Design of Efficient siRNA

When first published, DSIR was implemented through an interactive web platform (http://biodev.extra.cea.fr/DSIR/). In addition to the former models (i.e. 19- and 21-nt siRNA efficacy prediction), we have now added a model that corrects the predicted efficacy by a factor related to the location of the target site, in line with the observations presented here. Together with these computational models, the updated DSIR website also provides additional functionalities such as a fast off-targets search algorithm, 3′ UTR seed matches, a sequence filtering tool for specific motifs (e.g. immunostimulatory motifs, polynucleotide tracts), and export facilities to facilitate siRNA and shRNA sequence ordering. The DSIR website is freely available and regularly maintained by updating sequence databanks; it is continuously improved based on users’ feedback on usability and new functionalities. This has resulted in the website being widely used internationally (with an average of around 400 visits/month in 2011). Finally, this web resource provides supplementary materials (http://biodev.extra.cea.fr/DSIR/reference.html) including the present set of siRNA sequences targeting ten cancer-related genes as validated molecular tools for further experimental investigation. This dataset will be an additional resource for those seeking to benchmark siRNA design algorithms, and should stimulate future developments, it is complementary to the other datasets that have already been widely used in this context [40].

## Discussion

### 1 Experimental Analysis of DSIR Design Performance

Despite considerable and regular progress in siRNA design methods facilitating selection of functional siRNA the need for experimental validation has yet to be overcome. As mentioned above, most computational models to design siRNA were developed using heterogeneous datasets. This makes it difficult to assess how these predictive models perform in real-life. Because measuring the precise level of gene silencing for each siRNA is a demanding process (high-cost and time-consuming), and assays need to be finely tuned for newly targeted genes, reporter-based assays have been developed to speed up the identification of the most potent siRNA sequences [15], [41]. Although reporter-based activity has been said to correlate well with the efficacy of endogenous target depletion, this has yet to be experimentally proven.

In this study, we used the DSIR algorithm to design a total of 128 siRNA sequences, and then evaluated the efficiency of these siRNA sequences in a cell-based assay. We focused on ten cancer-related target genes, all of which are expressed at low levels. This expression level was chosen as it has been reported that low-abundance gene products are less amenable to siRNA-mediated knockdown [42]. Moreover, as some of these genes code for enzymes, knockdown must be very efficient to yield a clear phenotype. This is in contrast to structural protein targets, for which even slight knockdown can result in a phenotypic effect. To avoid bias due to measurement systems, siRNA knockdown efficiency was systematically measured on endogenously expressed genes applying a standardized validation procedure. This means that our data reflect natural characteristics such as structure and localization of a target mRNA in the cellular environment. Overall analysis during this study showed than 76 of the 128 siRNA designed induced more than a 70% decrease in target expression level. This indicates that DSIR software is a highly potent siRNA designer.

This study used HeLa cells for cellular assays. HeLa cells are easily transfectable, thus they help avoid transfection rate variations due to the cellular model. Interestingly, we obtained comparable results with the Bosc cell line (data not shown). We also tested some siRNA sequences, targeting CSNK2A1 and CSNK2A2 (si_031, si_034, si_041, si_043), in the human mammary epithelial cell line MCF10A, both at the mRNA and protein levels. These assays confirmed the results obtained on HeLa cells [43]. Taken together, these results show that the potency of siRNA designed with DSIR is stable in a range of cell lines.

We also observed that siRNA reagents designed with DSIR are efficient even at low concentrations (1 nM quantities). This will help significantly reduce the risk of off-target effects. Indeed, in the absence of clear rules to reduce or prevent OTE, using only low concentration represents an ideal means to overcome these unwanted effects in phenotypic studies. As an illustration, in previous studies, siRNA-mediated down-regulation of ERCC1 required up to 200 nM of siRNA, for less than a 70% decrease in both mRNA and protein after 48 h [44]. The experiments described here used ten-fold less siRNA, and achieved a similar decrease in ERCC1 expression with 8 out of 9 siRNA designed by DSIR software. This success rate suggests that using DSIR software might help prevent OTE.

Although satisfactory, with a relative overall success rate of 58%, our extinction results highlight a more complex picture, with different extinction profiles observed between target genes. Of the eight targets, five seem to be relatively easily silenced. In contrast, the remaining three, especially ERCC2 and HDAC6, are difficult to silence more than 70%. Since all these target genes are expressed at low levels, these results suggest that this property alone is not an indicator of successful silencing potential, as was previously observed [45]. Moreover, if siRNA sequences with known negative criteria (such as those harboring polynucleotide tracts or targeting the 5′UTR region of the target mRNA) are discarded, the success rate for siRNA designed with DSIR reaches a very satisfactory level for at least five of our target genes (CSNK2A2, ERCC1, CSNK2B, CSNK2A1, BCL2L1). The most satisfactory silencing was achieved with CSNK2A2, for which all siRNA efficiently knocked down expression. Meister and Rossi recommended designing five siRNA sequences per target to ensure at least one efficient siRNA [8], [46]. Based on the results presented here, this limit can be reduced. This will allow synthesis of a smaller number of reagents targeting most transcripts. However, this must be moderated by the success rate observed for ERCC2 and HDAC6. Results for these genes suggest that significant features involved in siRNA-mediated silencing remained to be identified.

### 2 Computational Analysis of the Main Features Potentially Contributing to siRNA Efficiency

This study provides a completely new dataset combining the siRNA sequences, and their predicted and experimentally measured efficiencies. This dataset constitutes a powerful tool to identify, analyze and validate potential features contributing to siRNA efficiency. Since the DSIR algorithm was designed based on analysis of intrinsic guide-strand sequence determinants on the siRNA, we further checked all determinants and descriptors that have been shown or are suspected to be involved in the RNAi pathway. As a rule, less attention has been paid to the intrinsic nature of the target. We therefore undertook a systematic evaluation of the influence of morphological mRNA features of the target site on silencing activity; these include untranslated transcribed regions, transcript length, number of exons, and exons boundaries. As expected, all the siRNA targeting the 5′UTR regions performed poorly. This part of transcripts should therefore be avoided as a location for the target sequence when designing siRNA. We also observed that the 3′ UTR seems to be a suitable target site for knockdown experiments, as observed previously [5], [47]. This observation suggests that the 3′UTR could be an appropriate target for short transcripts for which highly efficient siRNAs targeting the CDS cannot be identified [14].

In addition to location of the siRNA, both the secondary structure of the RNA and the RNA-binding proteins on target sites can influence accessibility to siRNA and thereby modulate siRNA-mediated regulation [10], [48], [49], [50]. To support this idea, it has been observed that the absence of translation is correlated with improved silencing, suggesting that activated RISC competes with specialized mRNA proteins for access to the mRNA target [51]. Although target accessibility has often been suggested to influence silencing efficiency, no clear picture has yet emerged. It is not possible to study this using reporter constructs and fusion transcripts. Moreover, one of the major hurdles in assessing target accessibility is the lack of tools reliably predicting secondary mRNA structure, not to mention the fact that in cells, mRNA is embedded in a ribonucleoprotein complex of unknown architecture. Indeed, although existing algorithms predicting RNA secondary structure are computationally efficient, each has inherent limitations making it difficult to estimate these criteria precisely. Here, we used the popular SFold server [49] to compute probability profiles for target accessibility for each of the 88 sites targeted by our siRNA. None of these profiles correlates clearly with the activity measured for a given siRNA (see supplementary Figure S2). We also noticed that the siRNA sequences generated by SFold present almost no overlap with our siRNA dataset (data not shown). It was recently reported that target accessibility alone computed using RNAplfold, just like any other descriptor assessed above, is not sufficient to reliably predict siRNA efficacy [22]; accessibility must be combined with conventional design criteria to improve siRNA efficacy. However, using this criterion computed by RNAplfold did not add to efficacy in our hands. Thus, even if target accessibility can not be excluded, its relative contribution to the overall interfering pathway remains to be experimentally proven (for example by performing RNase H mapping assays).

One of the most interesting findings of our study was the identification of a relationship between silencing efficacy and the position of the siRNA within the target gene. Analysis of the siRNA positions with regard to their relative efficiencies showed an estimated loss of potency of 1% per 100 bp as the siRNA position moves away from the 5′ end of the coding sequence. This was a general trend. It should, however, be nuanced in light of the difference observed between ERCC2 and HDAC6, for which an extended number of siRNA reagents was designed and the slight negative correlations observed with LIG1 and PARP1. To our knowledge, this is the first time that a position effect is reported. The molecular mechanisms responsible for this positional effect are not known. Indeed, the fact that this positional effect is detected only for some of the targets suggests a pathway dependent on sequence or molecular context rather than a more global effect. Obviously, this positional effect is not sufficient to fully explain all variations in siRNA efficiencies, but it may highlight some new, as yet unidentified, process regulating siRNA silencing. This will require further study to be fully characterized.

### Conclusion

In this paper, we experimentally validated the capacity of DSIR software to design siRNA by measuring actual knockdown efficiencies for siRNA with high predicted efficiencies. This analysis showed that DSIR estimates agree well with experimental silencing, confirming that DSIR is one of the best predictors of active siRNA [18]. Very recently, DSIR was also proven to reliably predict shRNAs for effective knockdown in transgenic flies [52]. Moreover, DSIR siRNA efficiency has been extensively validated on long term silenced human cells [55].

The new dataset presented here, containing over one hundred qualified siRNA sequences, will be helpful for the community working toward improved siRNA design which is in constant progress [40]. We have described how this dataset can be used to perform a comprehensive study of numerous features potentially contributing to silencing efficiency. Based on our observations and past lessons, we updated our interactive DSIR Web tool to improve siRNA design. This web tool and the list of validated siRNA directed against very relevant cancer-related targets are freely accessible.

## Supporting Information

Figure S1 **Molecular analysis of gene silencing for seven targets.** HeLa cells were transfected with siRNA against the target genes indicated, and with two control siRNA, (GFP as a negative control and CSNK2B as a positive control). All siRNA were used at a final concentration of 20 nM, and were transfected using Oligofectamine reagent. Cells were also mock transfected (without siRNA). Three days later, RNA and proteins were extracted for further analysis. **A.** Effect of siRNA treatment from a typical experiment. For each siRNA the relative quantity of the target mRNA to HPRT (black) or 36B4 (grey) was plotted using the comparative analysis module in MxPro software (Stratagene). **B.** Transfection efficiency control. For each experiment, transfection efficiencies were checked by quantifying gene silencing relative to a control siRNA of known efficiency. Results of experiments where this control did not silence expression by more than 70% were excluded from the dataset because transfection efficiency was considered to be poor. **C.** Box plot representation of siRNA efficiency for 10 sequences. For each siRNA, efficiency predicted by DSIR and measured efficiency are indicated. Measured efficiency was statistically determined from triplicate RT-qPCR quantification of target mRNA after siRNA treatment, based on three independent experiments. Expression levels were normalized to HPRT (black) and 36B4 (red) house-keeping genes. Log(Q) = 1 represents no reduction in target mRNA after treatment and log(Q) = 1/4 equates to approximately 75% efficiency. See section 2.6 for further details of the statistical analysis. Overall siRNA efficiency and significance values are provided in supplementary material. Each panel corresponds to one target gene: ERCC1, CSNK2A2, CSNK2B, HIF1A, HDAC6, ERCC2 and BCL2L1.
(PDF)

Figure S2 **Target accessibility prediction profile for the eight mRNA targets and 88 corresponding siRNA sequences.** Each full-length sequence target was submitted to the SFold server (siRNA section - http://sfold.wadsworth.org/cgi-bin/sirna.pl). The target accessibility probability profile for each site targeted by the siRNA is displayed. Blue circle highlights target sites for a given siRNA guide strand. For each siRNA, information in the box indicates: its identifier, start and end positions in the target and the knockdown activity measured (in bold red).
(PDF)

Table S1 **Total set of 128 siRNA sequences.** Position in full-length transcript are given in bp relative to the 5′ extremity. SS sequence means sense strand siRNA sequence, in 5′ to 3′ orientation. AS sequence means antisense strand siRNA sequence (guide strand), in 5′ to 3′ orientation. DSIR corresponds to the efficacy score computed by the 21-nt linear model.
(XLS)

Table S2 **qPCR primer sequences used in this study.**
(XLS)

Table S3 **Features computed from the total set of siRNA sequences**. siRNA_id: siRNA identifier; Target Length: full length in nucleotides; #Exon: number of exons in the target (as documented by the RefSeq division of the NCBI database, release 48); Target Position (in full-length): starting position of the region targeted by the siRNA (antisense strand); DSIR score: siRNA efficacy predicted by the DSIR computational model; %silencing (from dilution series): % silencing for each siRNA expressed as the percentage of residual non-cleaved mRNA relative to control, determined by the dilution series methods (see materials & methods and supplementary material); Accessibility (RNAplfold): probability of target accessibility, computed by the RNAplfold program; #Off-target: number of potential off-targets based on screening against RefSeq with a mismatch tolerance of 3; #Seqs: number of 3′UTR sequence regions matched; #Seed hit1: total number of seed sites (encompassing positions 2 to 8 of the guide strand) matching a 3′UTR sequence region only once; #Seed hit2: number of seed sites matching a 3′UTR sequence regions twice; #Seed hit3+: number of seeds matching a 3′UTR sequence regions three (or more) times; Location: part of the transcript region targeted (5′UTR, CDS or 3′UTR); polyN >4: indicates a sequence of four (or more) identical nucleotides in the guide strand; Target exon lengh siRNA: length of the exon targeted by the siRNA sequence; siRNA exon mapping: siRNA overlapping exon-exon junction target sites (0 for no overlap, 1 for overlap); %silencing (from statistical model):extinction values for each siRNA calculated using the statistical model (see supplementary material).
(XLS)

Materials S1 **Materials and References.**
(DOC)
