# Supplementary material for: DSIR: Assessing the Design of Highly Potent siRNA by Testing a Set of Cancer-Relevant Target Genes
Source: PLoS One. 2012 Oct 30;7(10):e48057. doi: 10.1371/journal.pone.0048057 (PMC3484153; doi:10.1371/journal.pone.0048057)

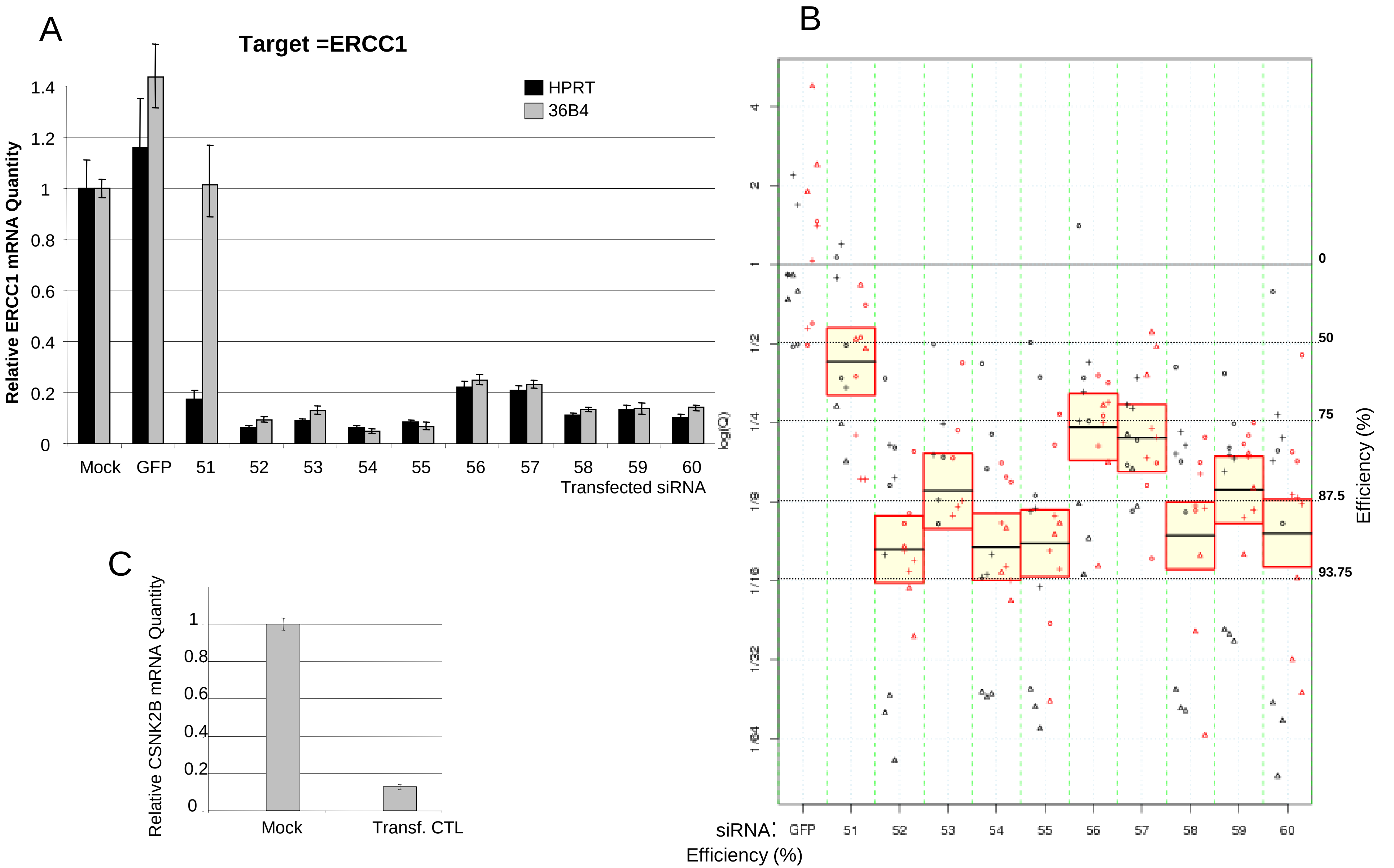

**Predicted**	**101**	**96**	**89**	**89**	**88**	**86**	**83**	**83**	**82**	**U**
**Measured**	**57**	**92**	**86**	**92**	**91**	**76**	**78**	**90**	**86**	**90**

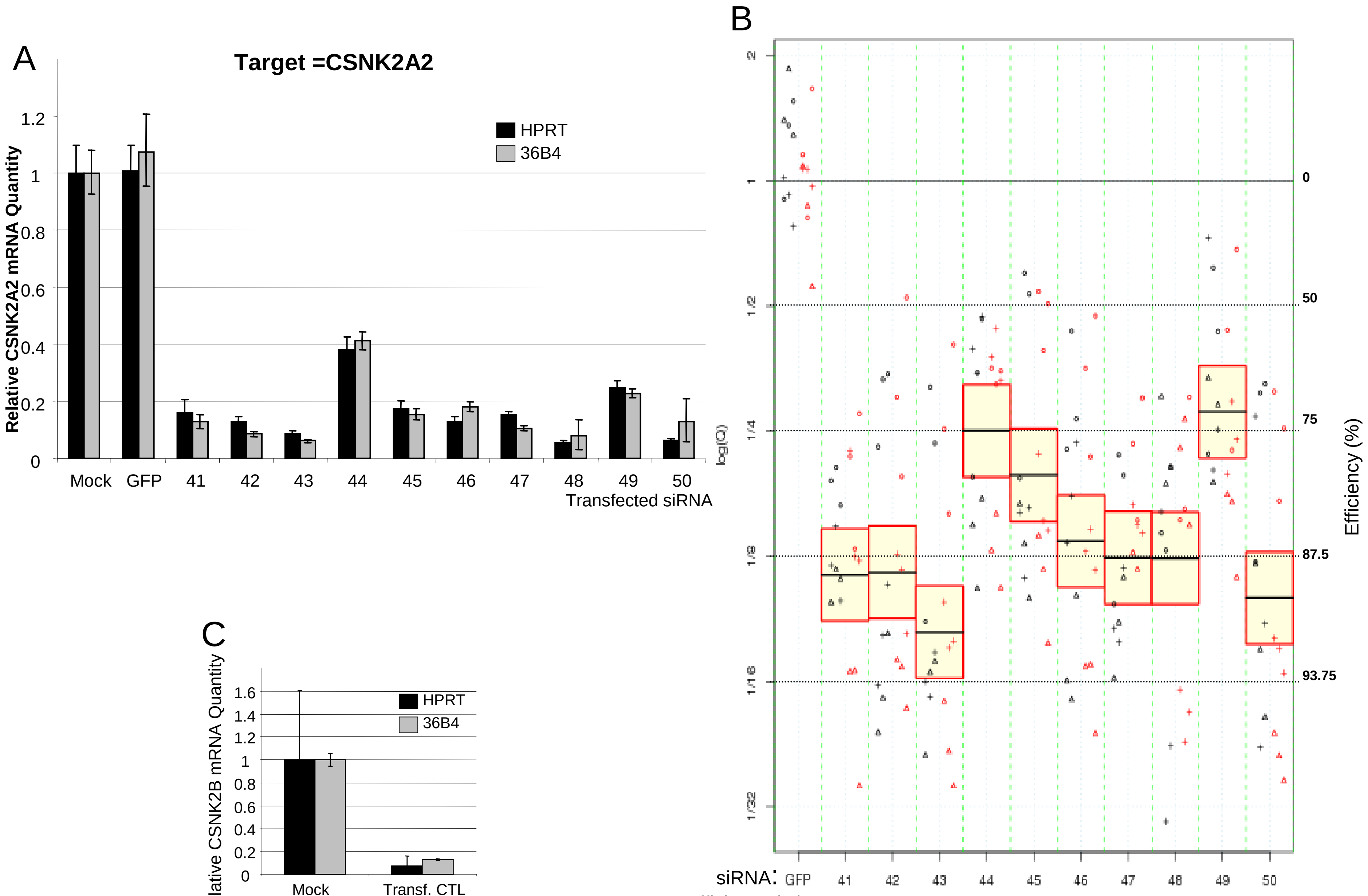

Efficiency (%)

**Predicted**	**96**	**92**	**85**	**84**	**84**	**83**	**82**	**81**	**81**	**80**
**Measured**	**89**	**88**	**92**	**75**	**80**	**86**	**87**	**88**	**72**	**90**

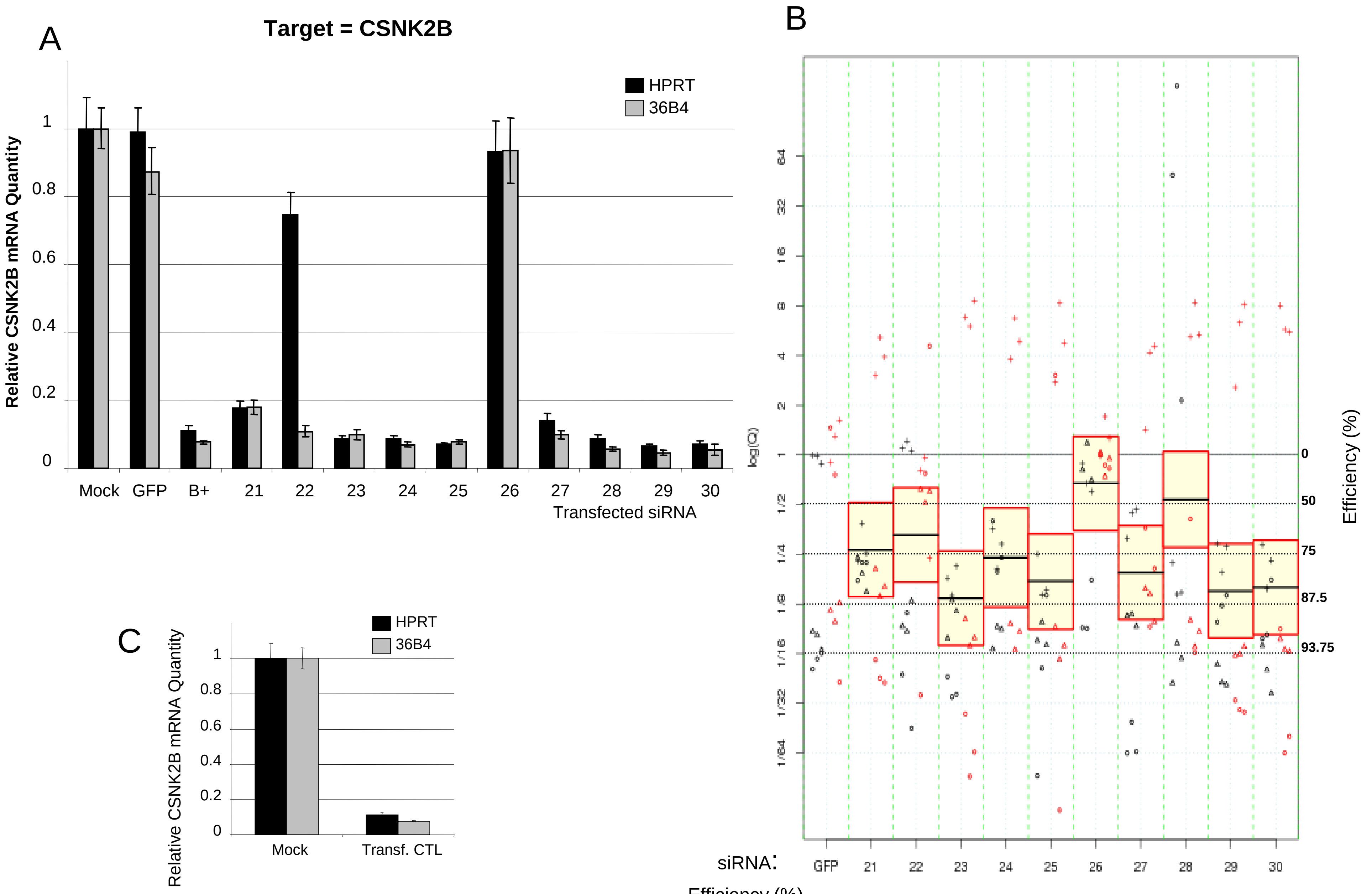

Efficiency (%)

**Predicted**	**95**	**91**	**89**	**86**	**86**	**85**	**84**	**83**	**82**	**82**
**Measured**	**83**	**60**	**92**	**76**	**89**	**34**	**85**	**62**	**91**	**92**

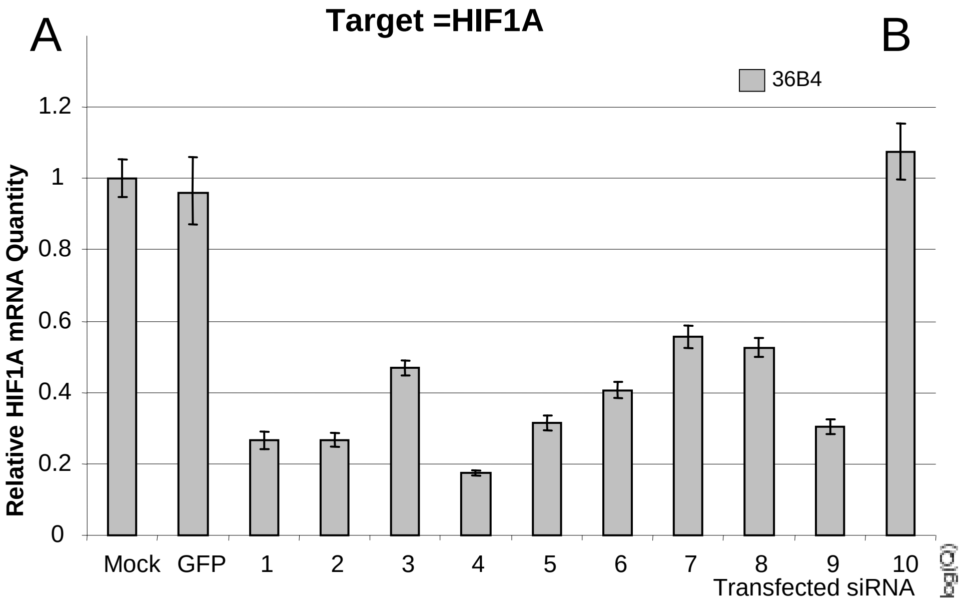

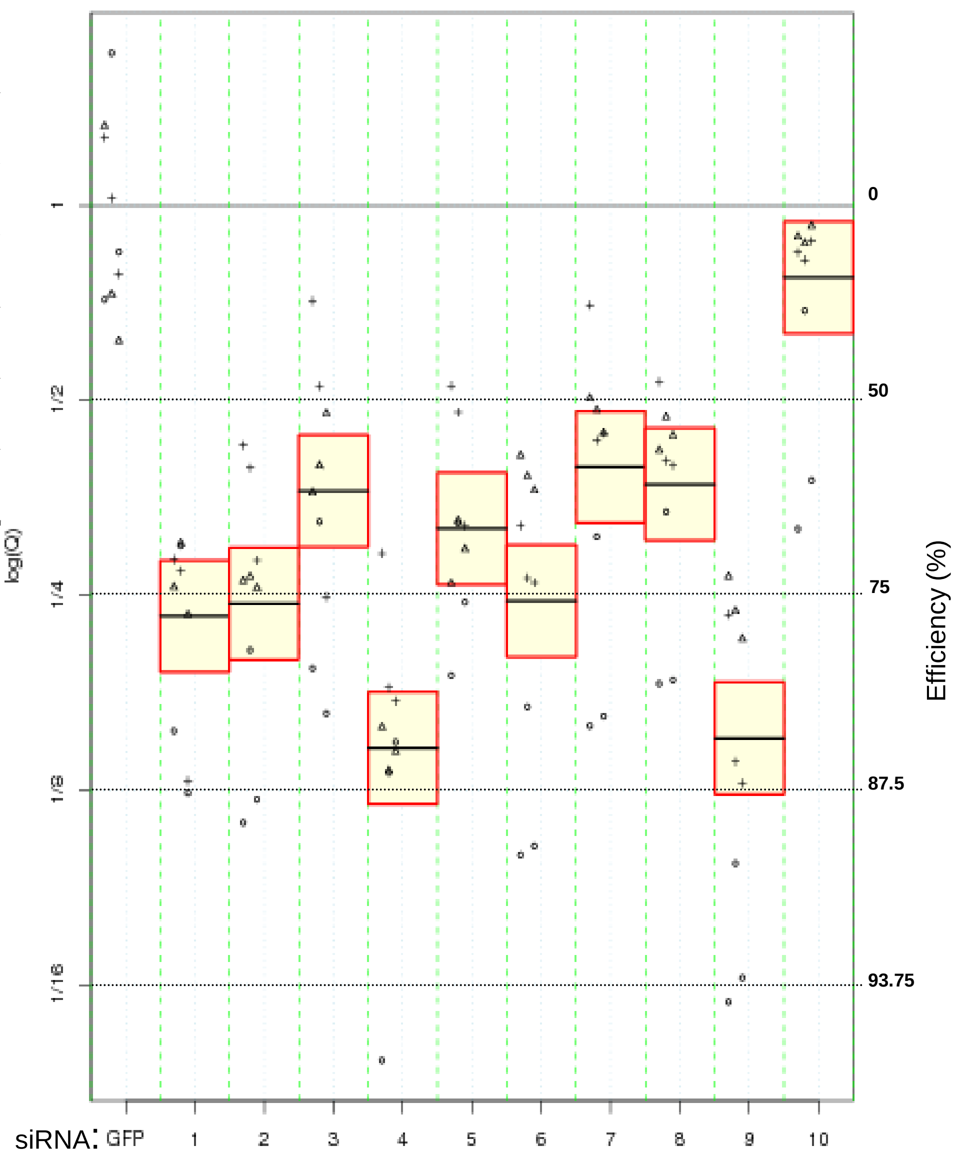

Efficiency (%)

**Predicted**	**101**	**99**	**97**	**94**	**94**	**91**	**87**	**87**	**85**	**38**
**Measured**	**77**	**76**	**64**	**86**	**68**	**76**	**61**	**63**	**85**	**23**

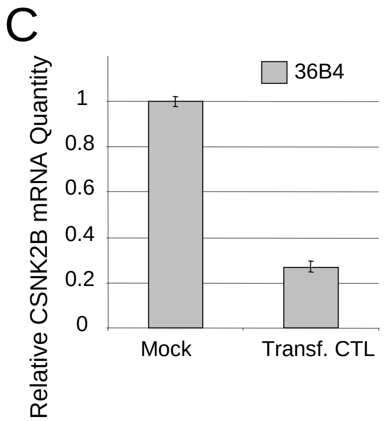

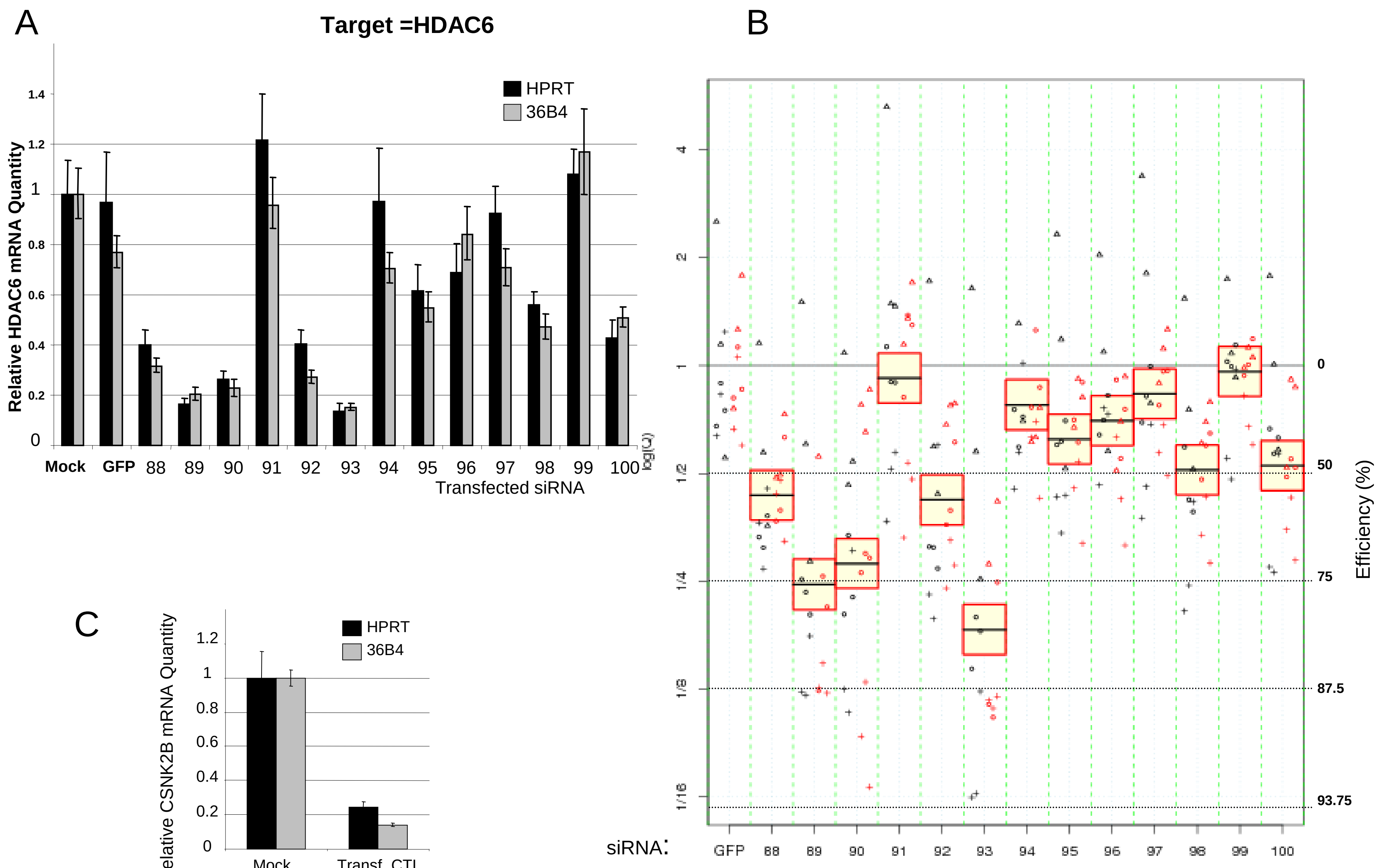

Efficiency (%)

**Predicted**	**100**	**100**	**100**	**98**	**96**	**95**	**94**	**94**	**90**	**87**	**92**	**95**	**88**
**Measured**	**57**	**72**	**67**	**11**	**56**	**82**	**21**	**35**	**25**	**11**	**47**	**3**	**44**

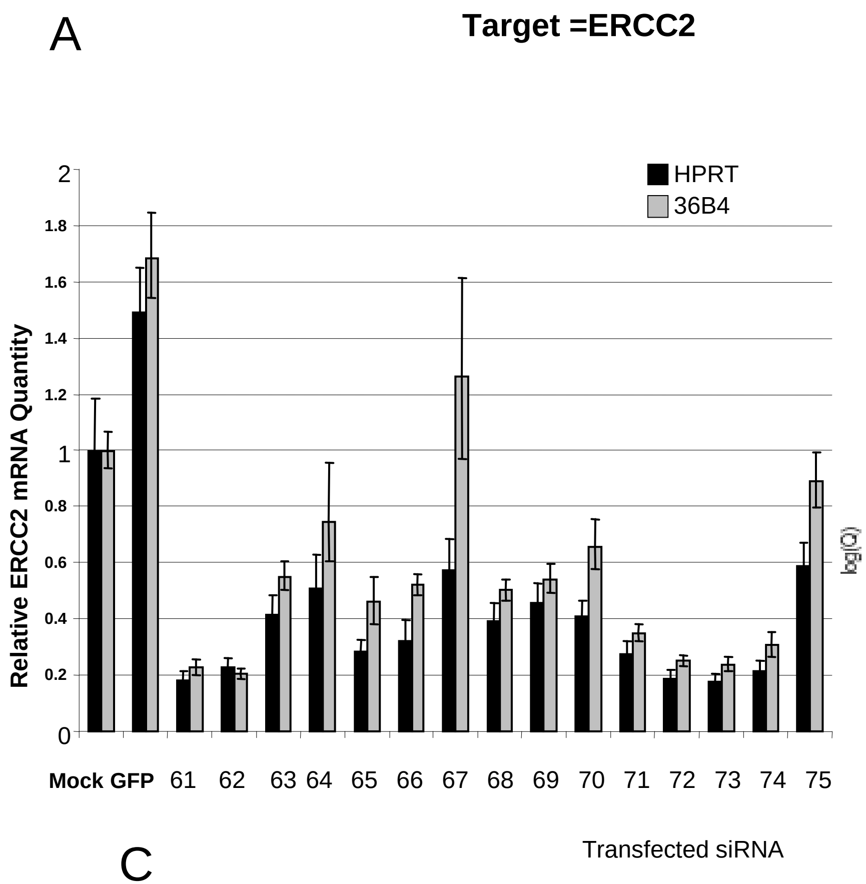

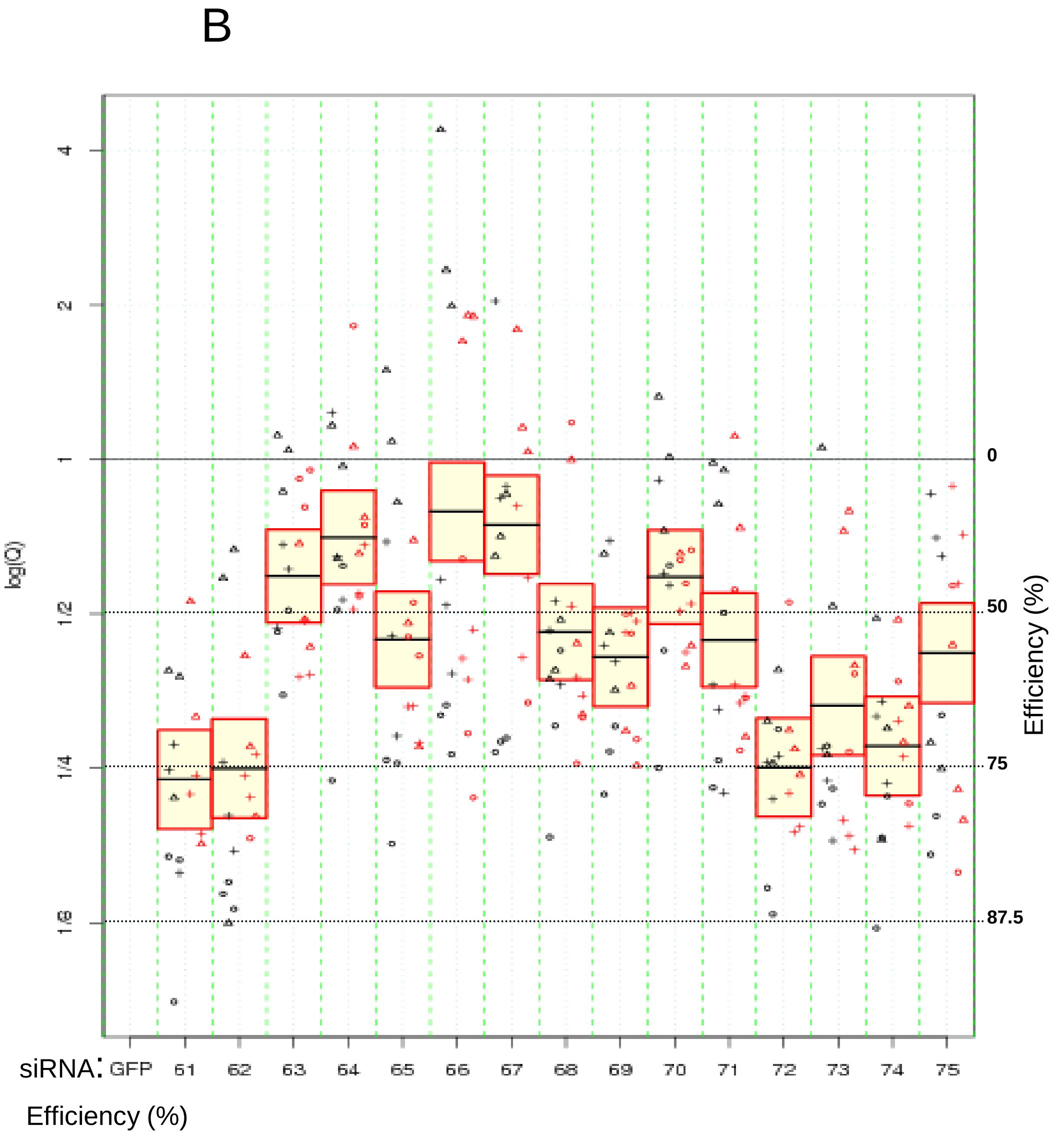

**Predicted**	103	82	86	80	82	92	91	87	82	88	86	88	85	83	89
**Measured**	78	76	46	35	58	23	27	57	60	45	56	76	67	73	60

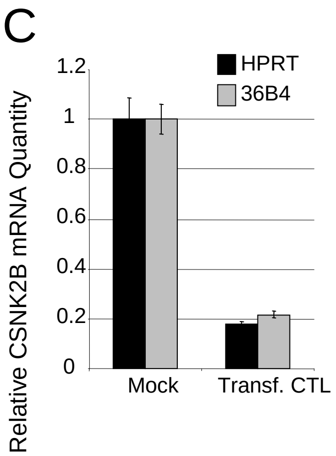

A

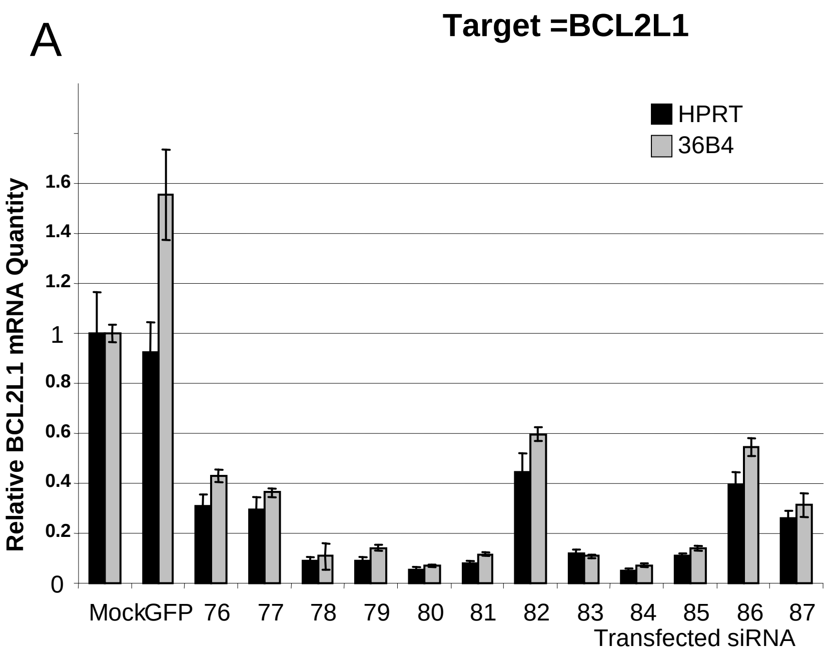

B

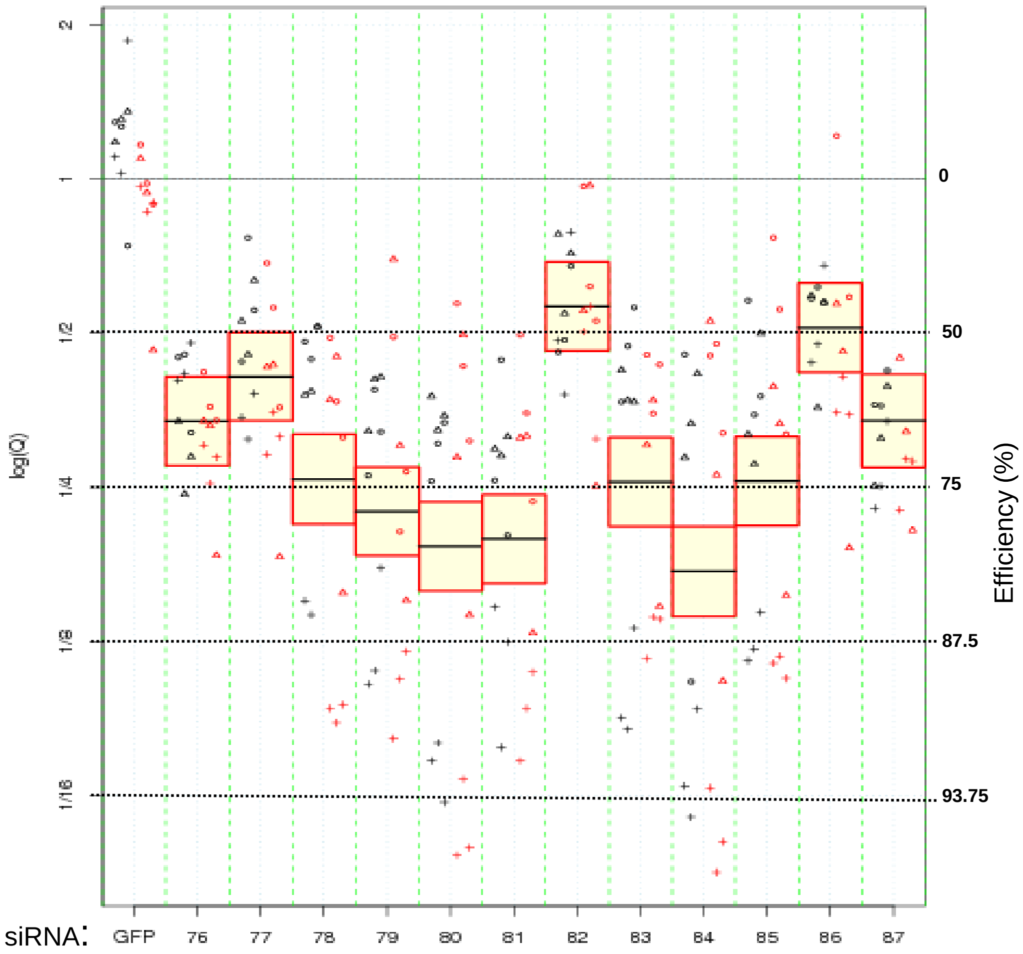

Efficiency (%)

**Predicted**	92	90	85	84	93	88	81	89	93	90	98	94
**Measured**	66	59	74	77	81	80	43	74	82	74	47	62

C

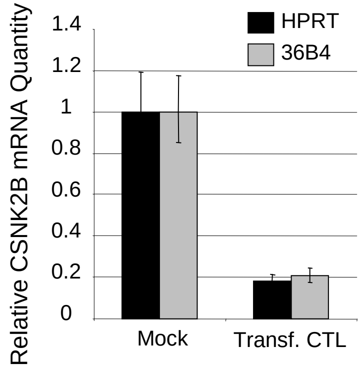

Supplement: Figure S1 — Molecular analysis of gene silencing for seven targets. HeLa cells were transfected with siRNA against the target genes indicated, and with two control siRNA, (GFP as a negative control and CSNK2B as a positive control). All siRNA were used at a final concentration of 20 nM, and were transfected using Oligofectamine reagent. Cells were also mock transfected (without siRNA). Three days later, RNA and proteins were extracted for further analysis. A. Effect of siRNA treatment from a typical experiment. For each siRNA the relative quantity of the target mRNA to HPRT (black) or 36B4 (grey) was plotted using the comparative analysis module in MxPro software (Stratagene). B. Transfection efficiency control. For each experiment, transfection efficiencies were checked by quantifying gene silencing relative to a control siRNA of known efficiency. Results of experiments where this control did not silence expression by more than 70% were excluded from the dataset because transfection efficiency was considered to be poor. C. Box plot representation of siRNA efficiency for 10 sequences. For each siRNA, efficiency predicted by DSIR and measured efficiency are indicated. Measured efficiency was statistically determined from triplicate RT-qPCR quantification of target mRNA after siRNA treatment, based on three independent experiments. Expression levels were normalized to HPRT (black) and 36B4 (red) house-keeping genes. Log(Q) = 1 represents no reduction in target mRNA after treatment and log(Q) = 1/4 equates to approximately 75% efficiency. See section 2.6 for further details of the statistical analysis. Overall siRNA efficiency and significance values are provided in supplementary material. Each panel corresponds to one target gene: ERCC1, CSNK2A2, CSNK2B, HIF1A, HDAC6, ERCC2 and BCL2L1. (PDF) [file pone.0048057.s001.pdf]
