# Supplementary material for: DSIR: Assessing the Design of Highly Potent siRNA by Testing a Set of Cancer-Relevant Target Genes
Source: PLoS One. 2012 Oct 30;7(10):e48057. doi: 10.1371/journal.pone.0048057 (PMC3484153; doi:10.1371/journal.pone.0048057)

SFOLD probability profiling for target accessibility prediction : HDAC6

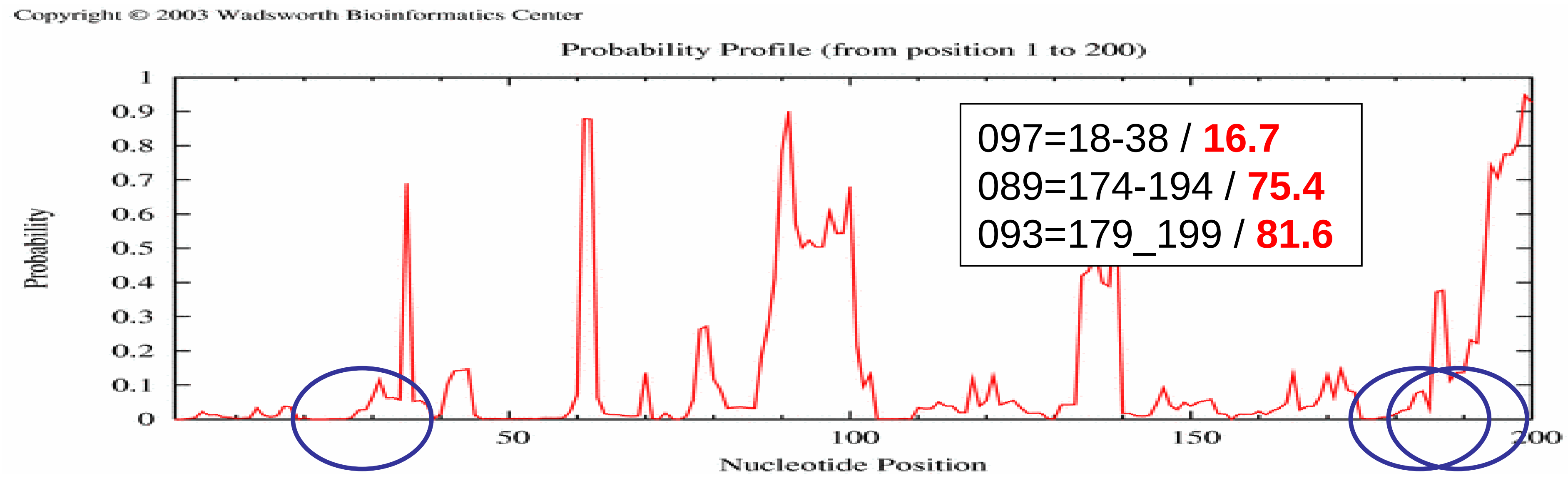

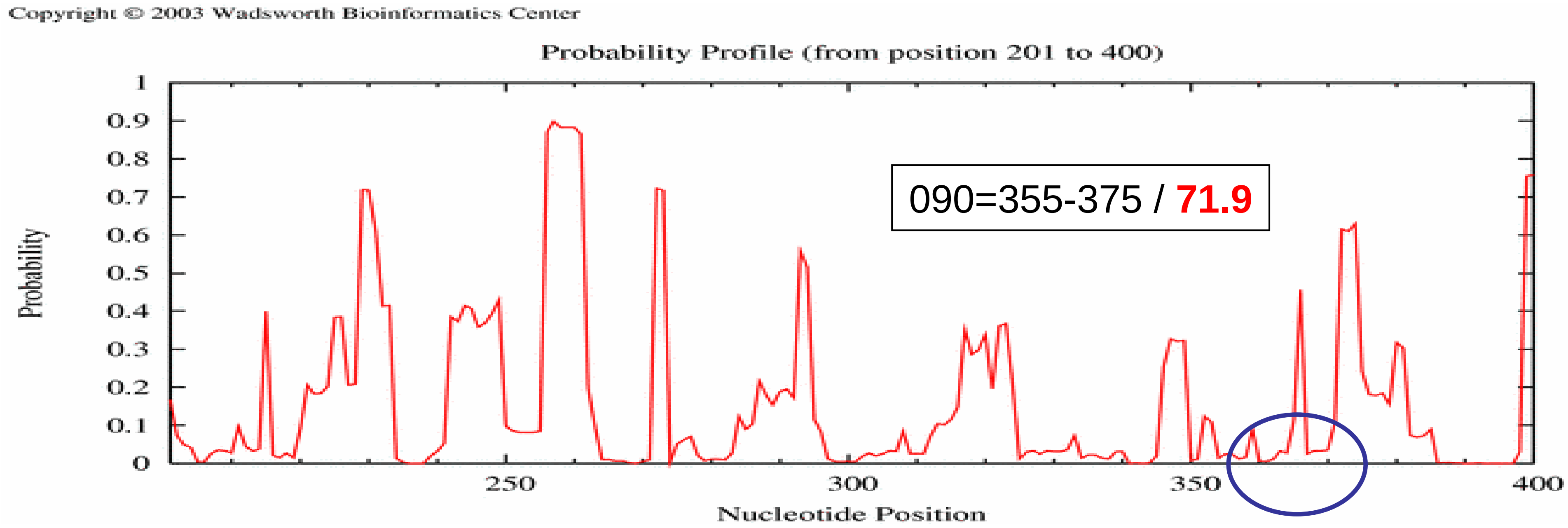

SFOLD probability profiling for target accessibility prediction : HDAC6

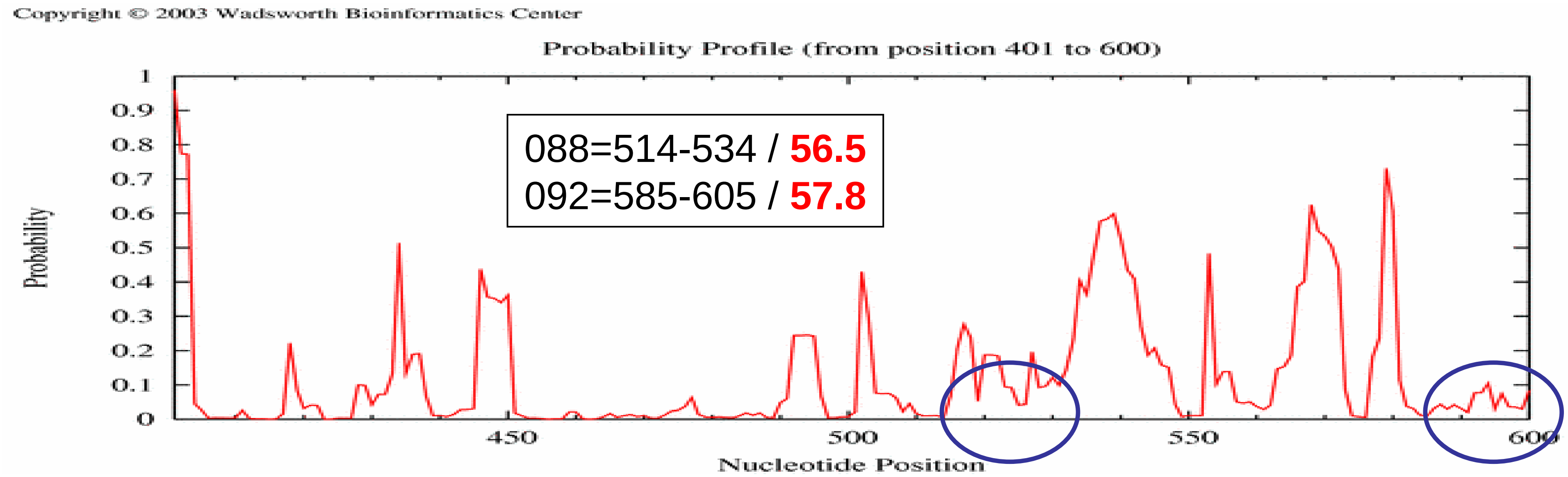

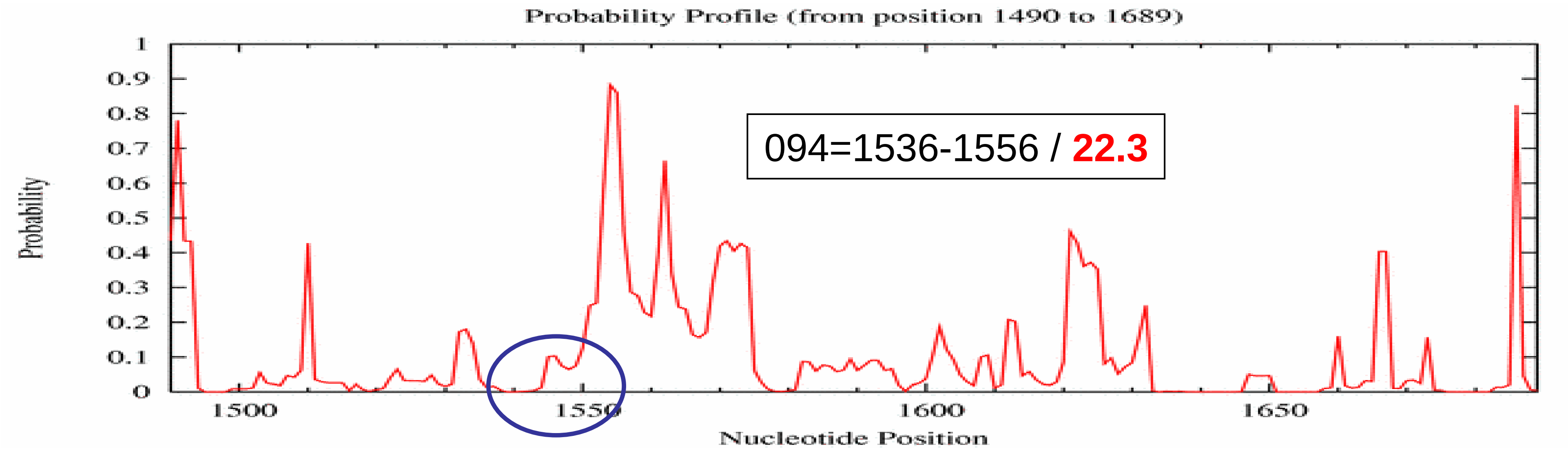

SFOLD probability profiling for target accessibility prediction : HDAC6

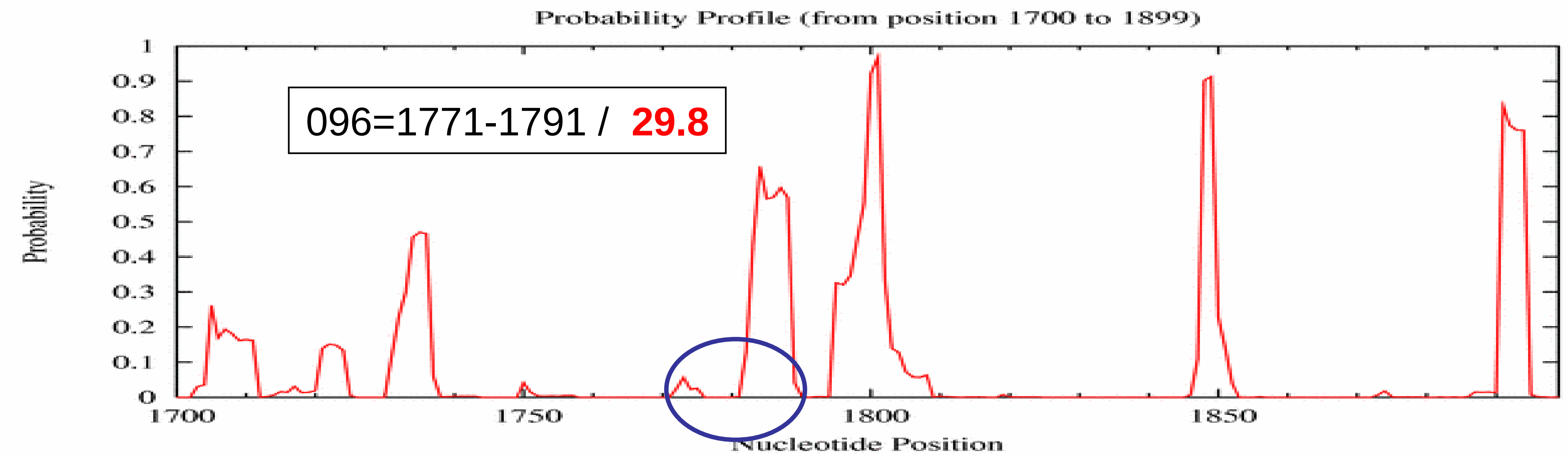

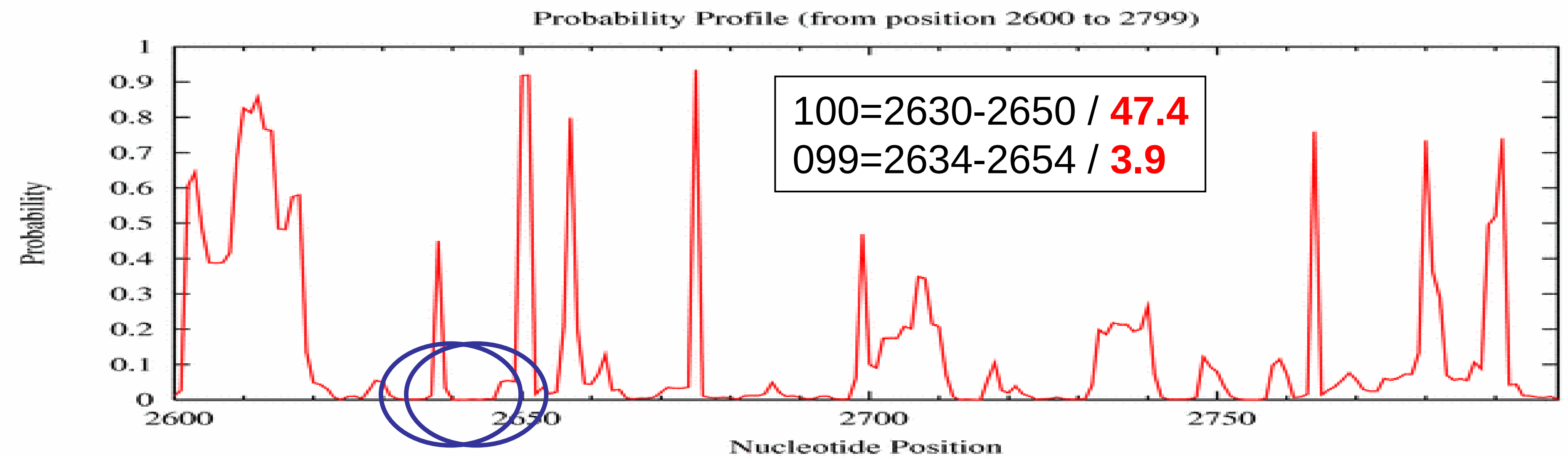

SFOLD probability profiling for target accessibility prediction : HDAC6

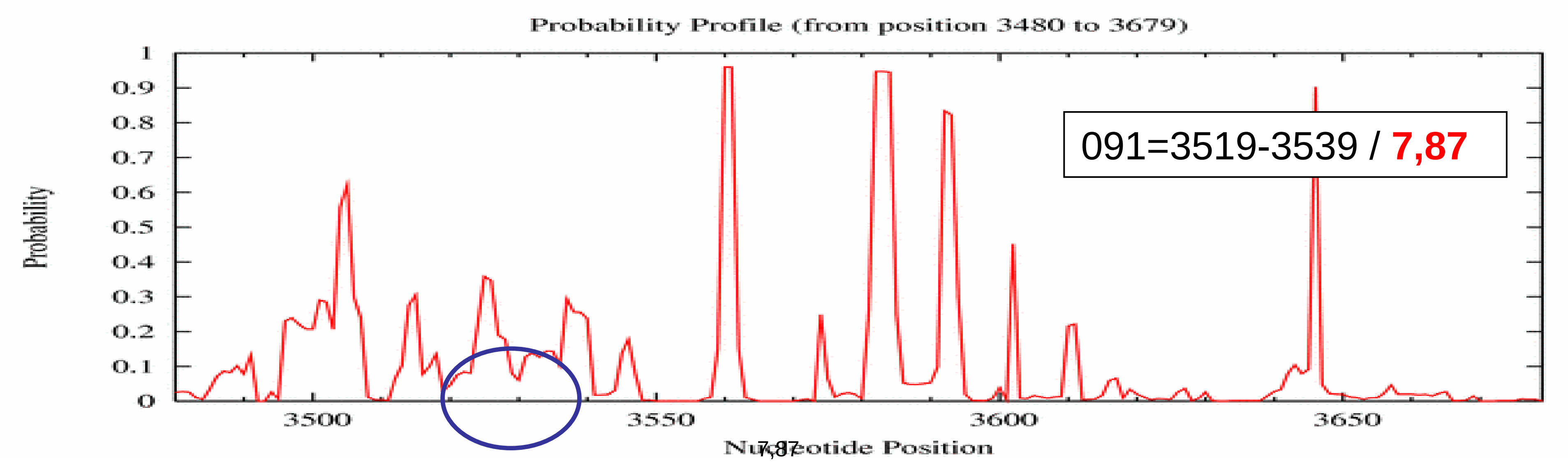

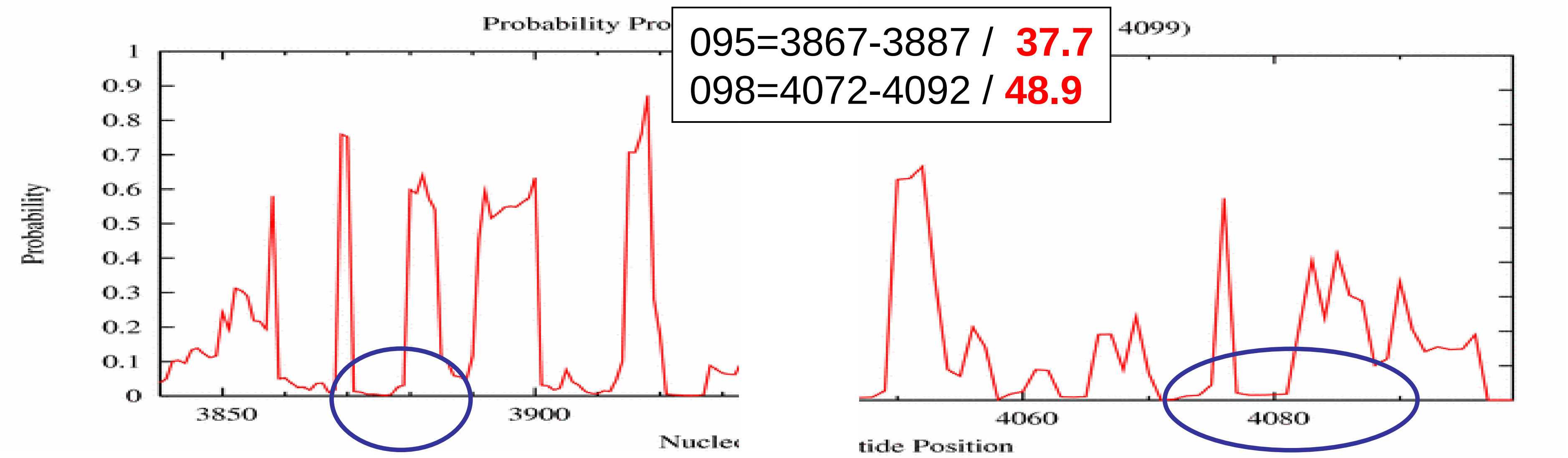

SFOLD probability profiling for target accessibility prediction : ERCC2

069=22-42 / **58.8**
078=28-48 / **53.9**

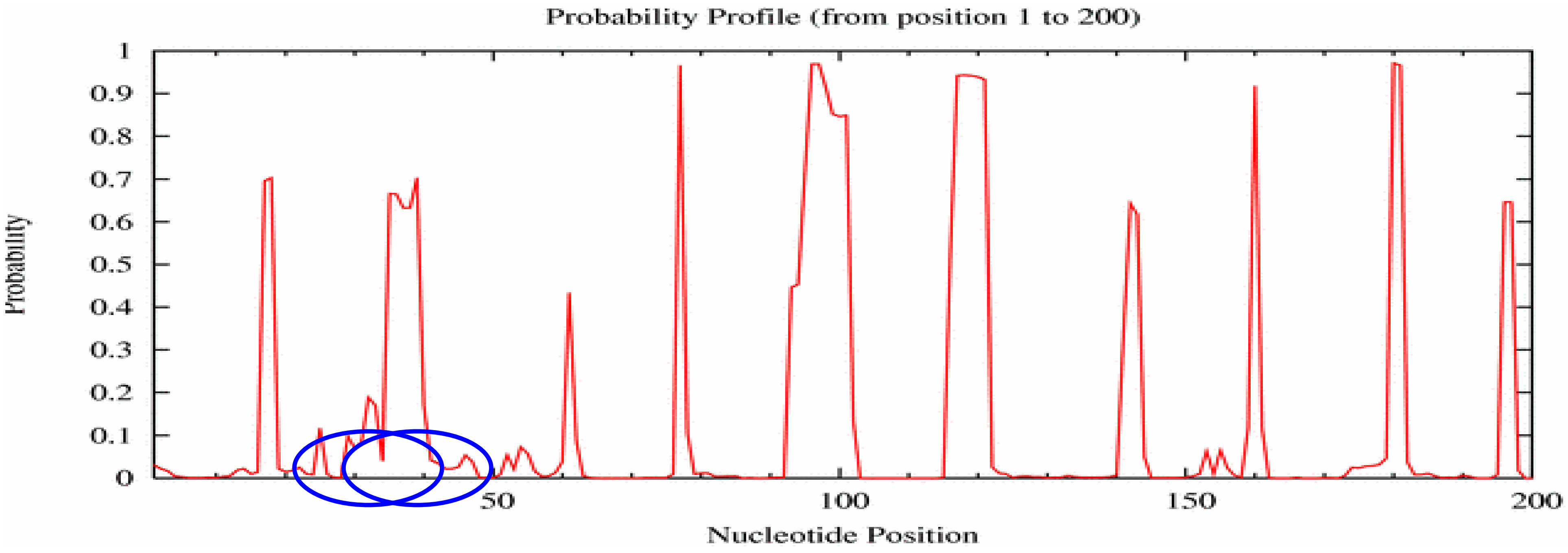

SFOLD probability profiling for target accessibility prediction : ERCC2

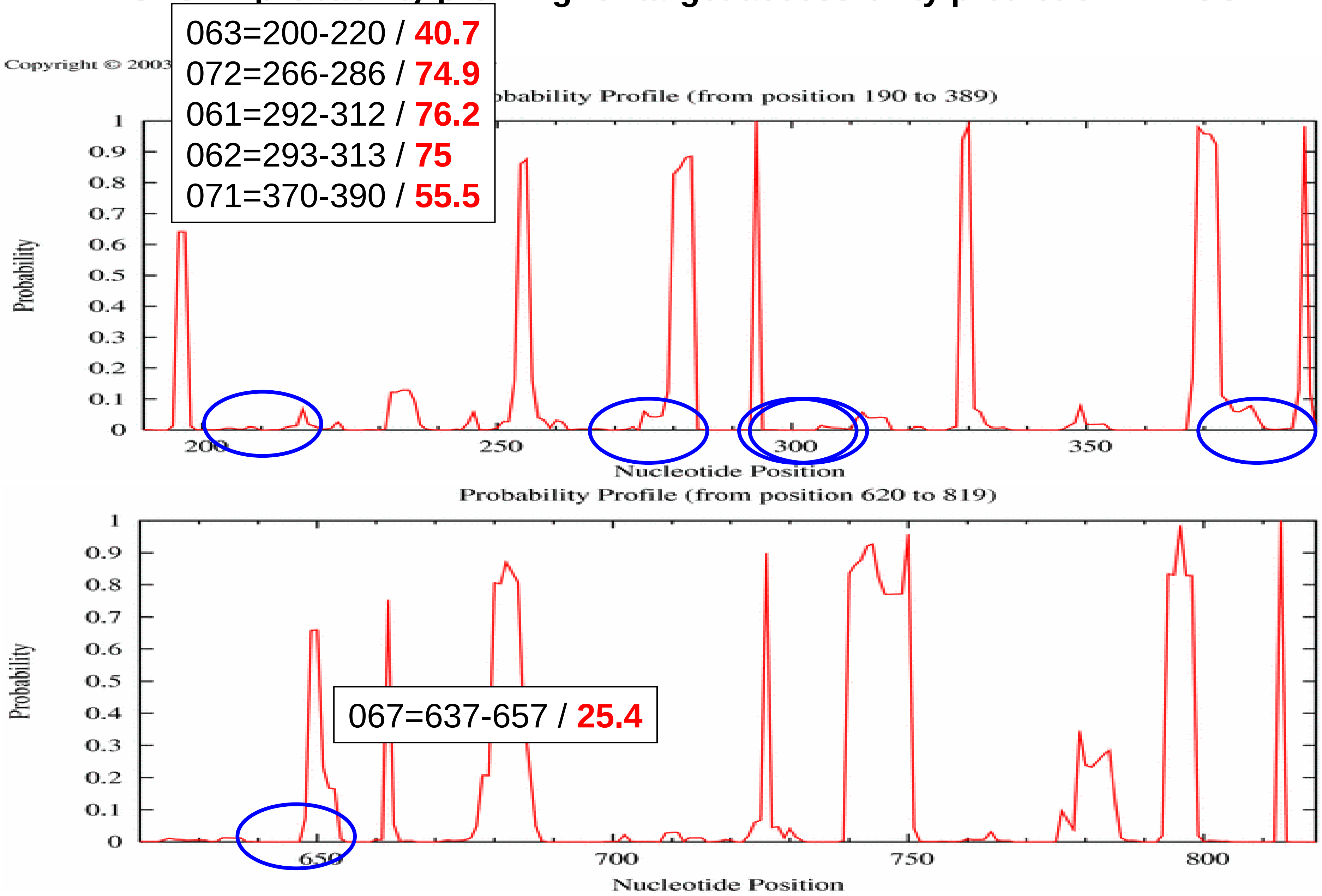

SFOLD probability profiling for target accessibility prediction : ERCC2

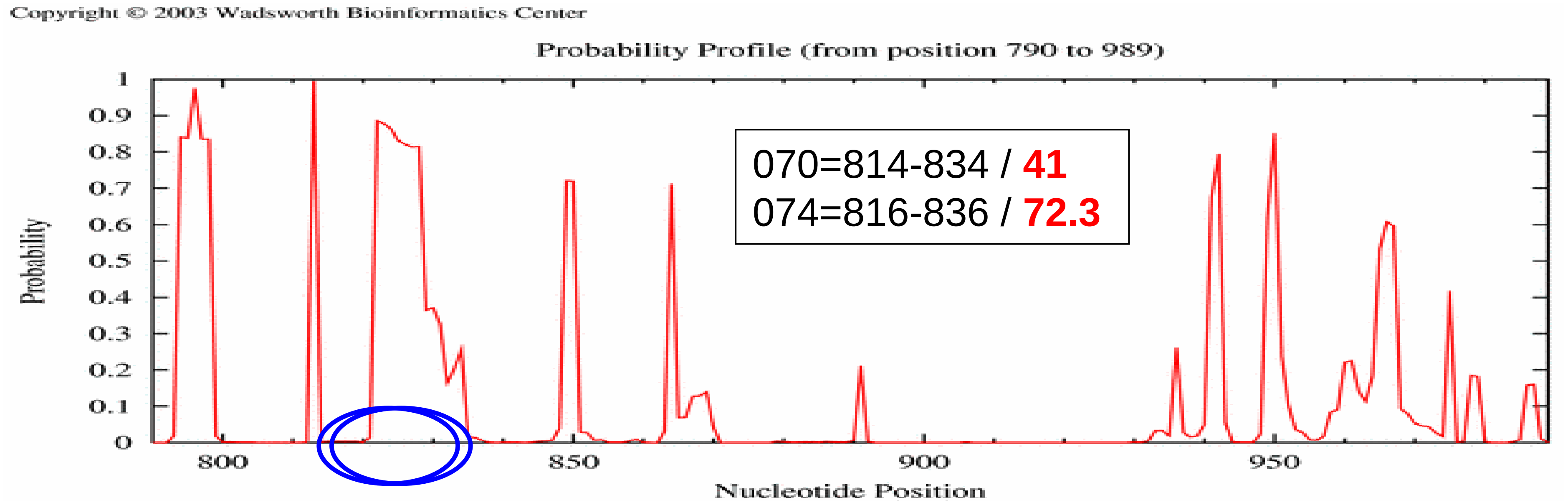

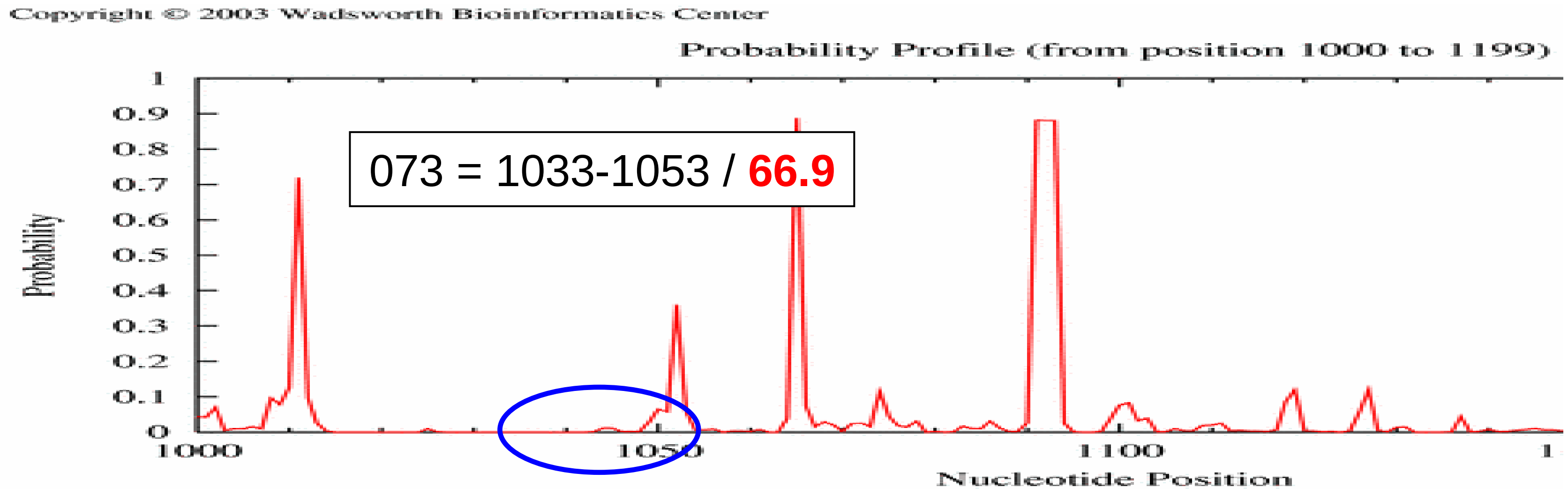

SFOLD probability profiling for target accessibility prediction : ERCC2

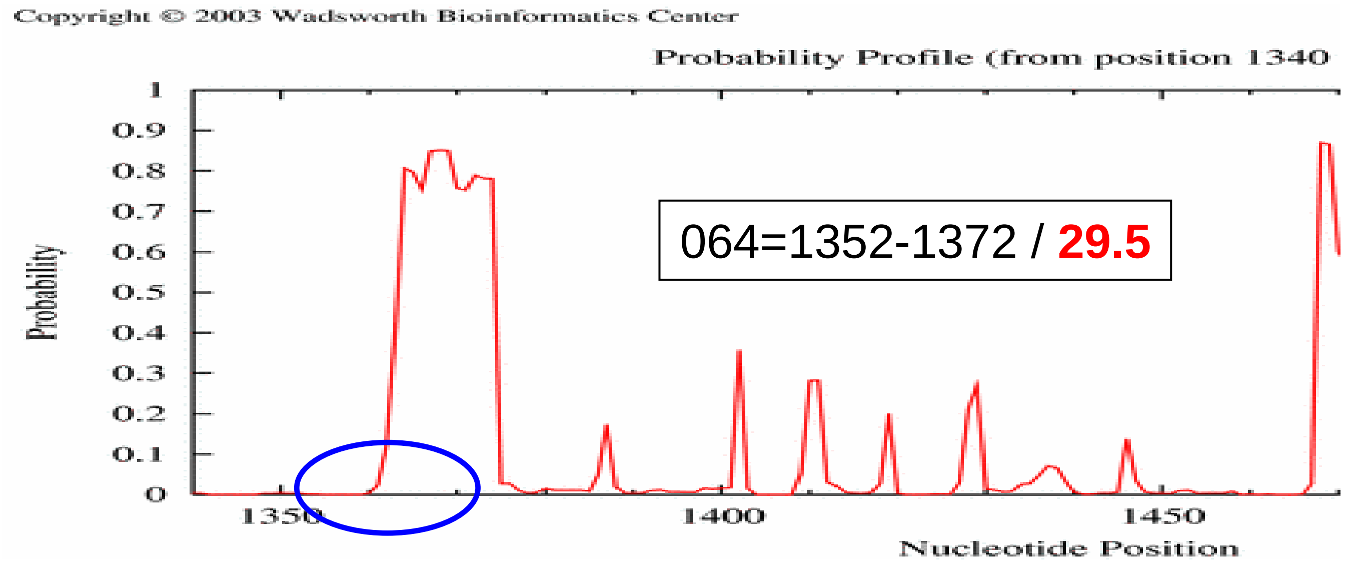

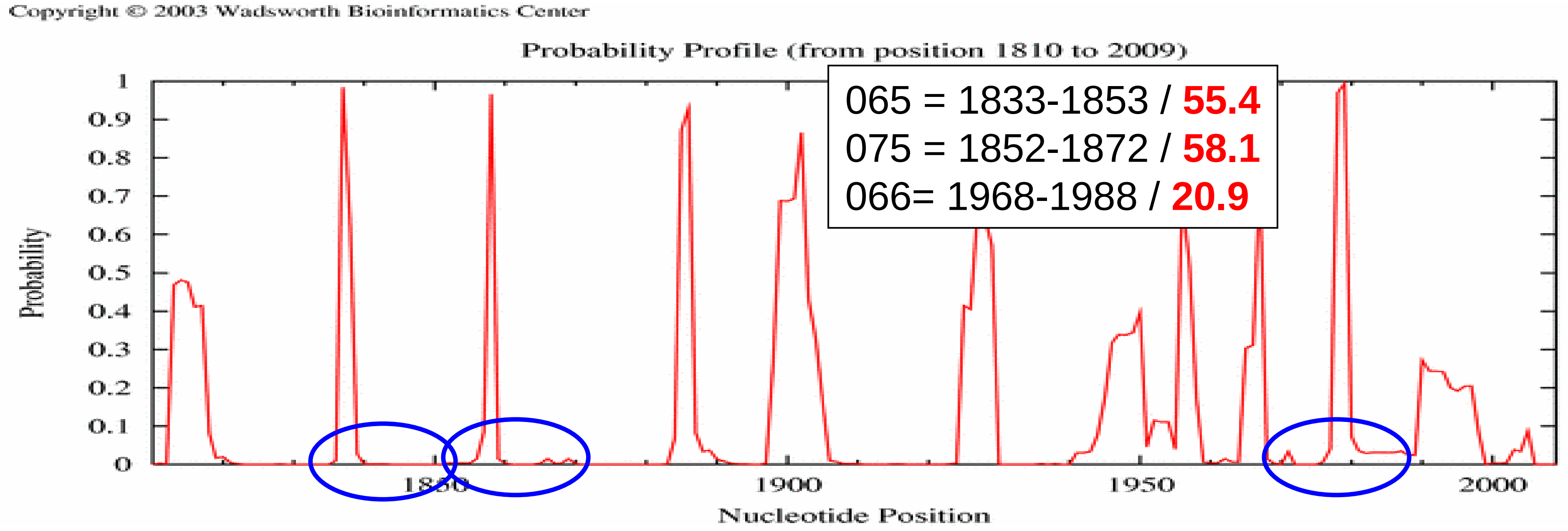

SFOLD probability profiling for target accessibility prediction : BCL2L1

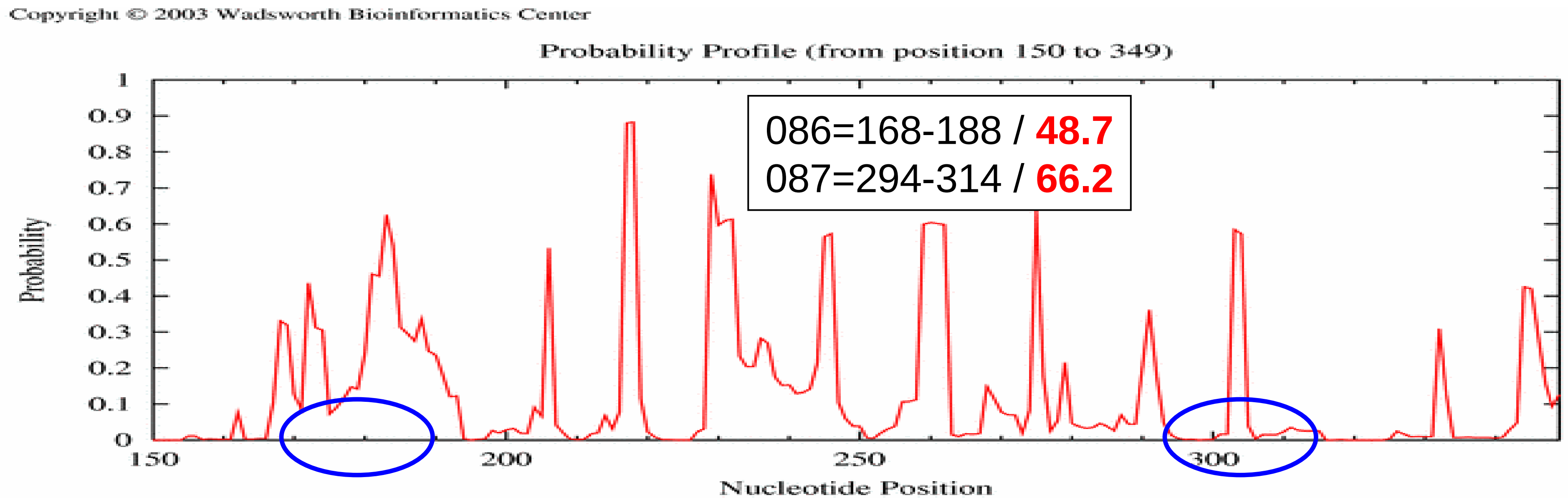

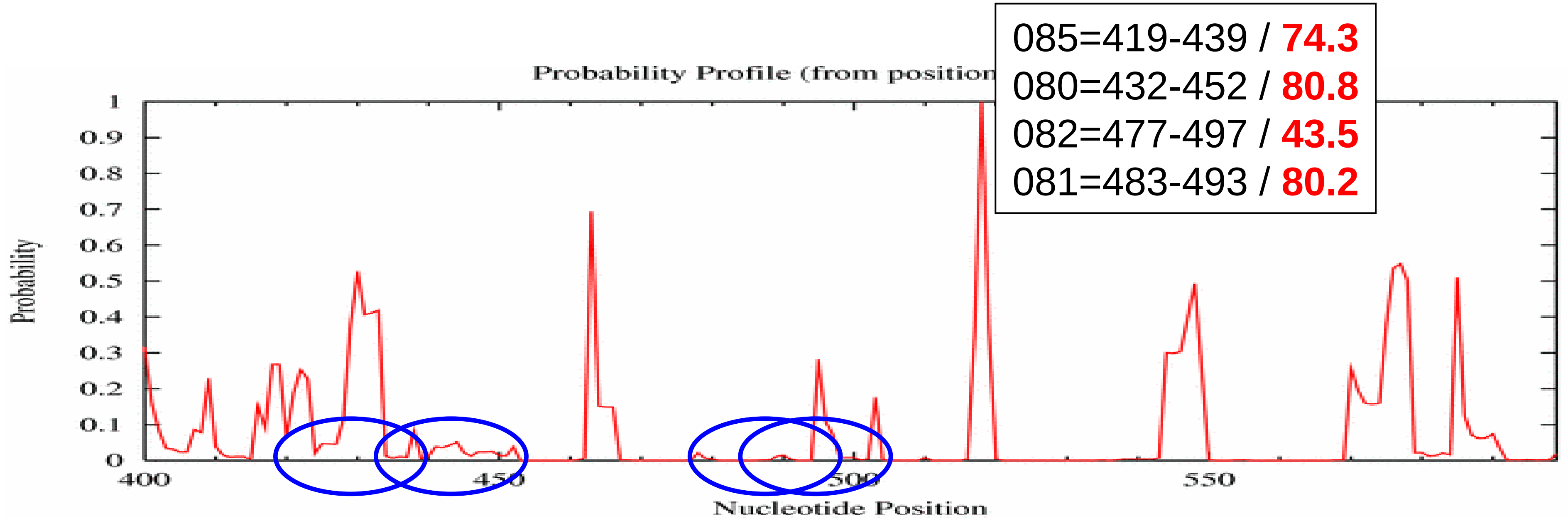

SFOLD probability profiling for target accessibility prediction : BCL2L1

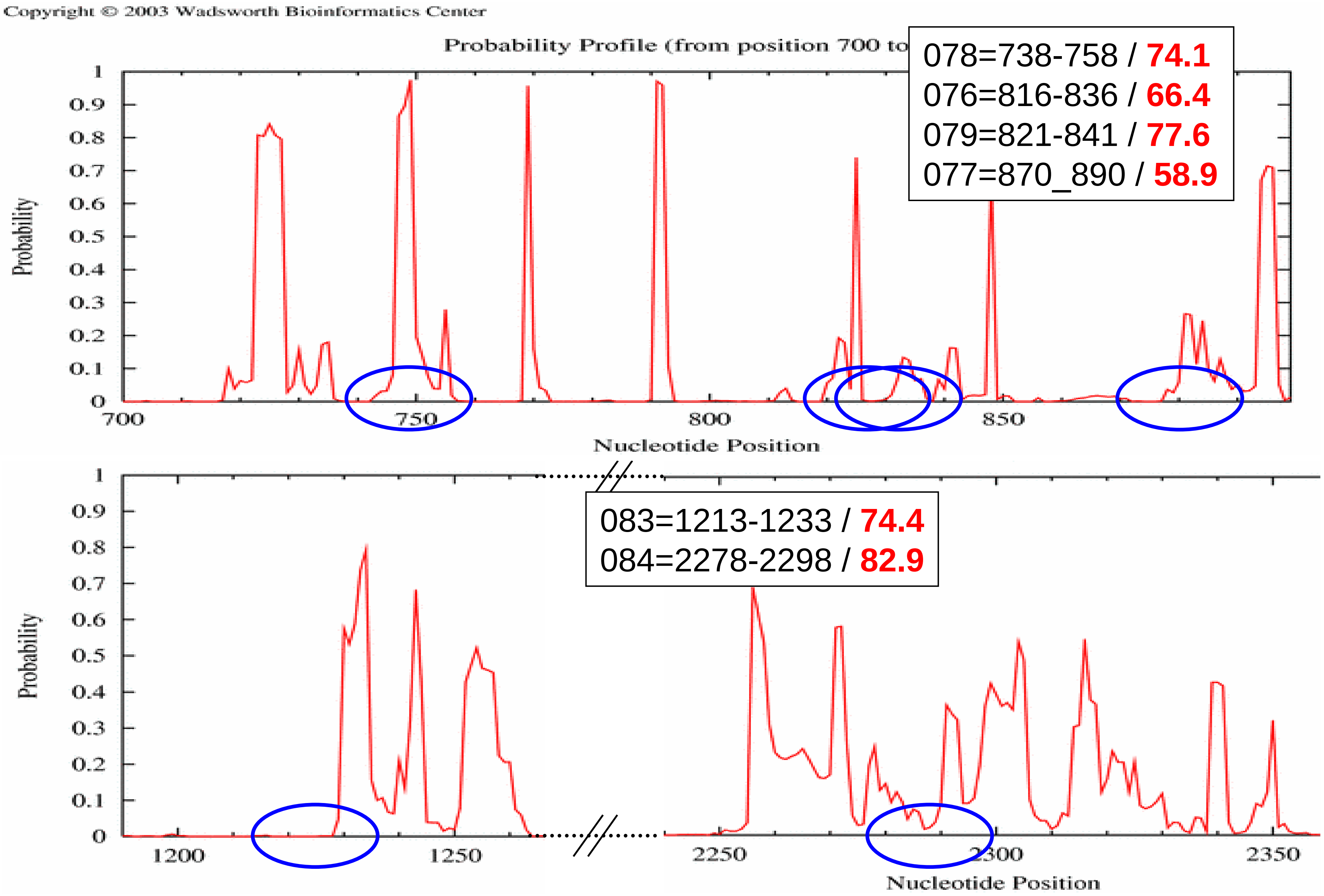

SFOLD probability profiling for target accessibility prediction : CSNK2A2

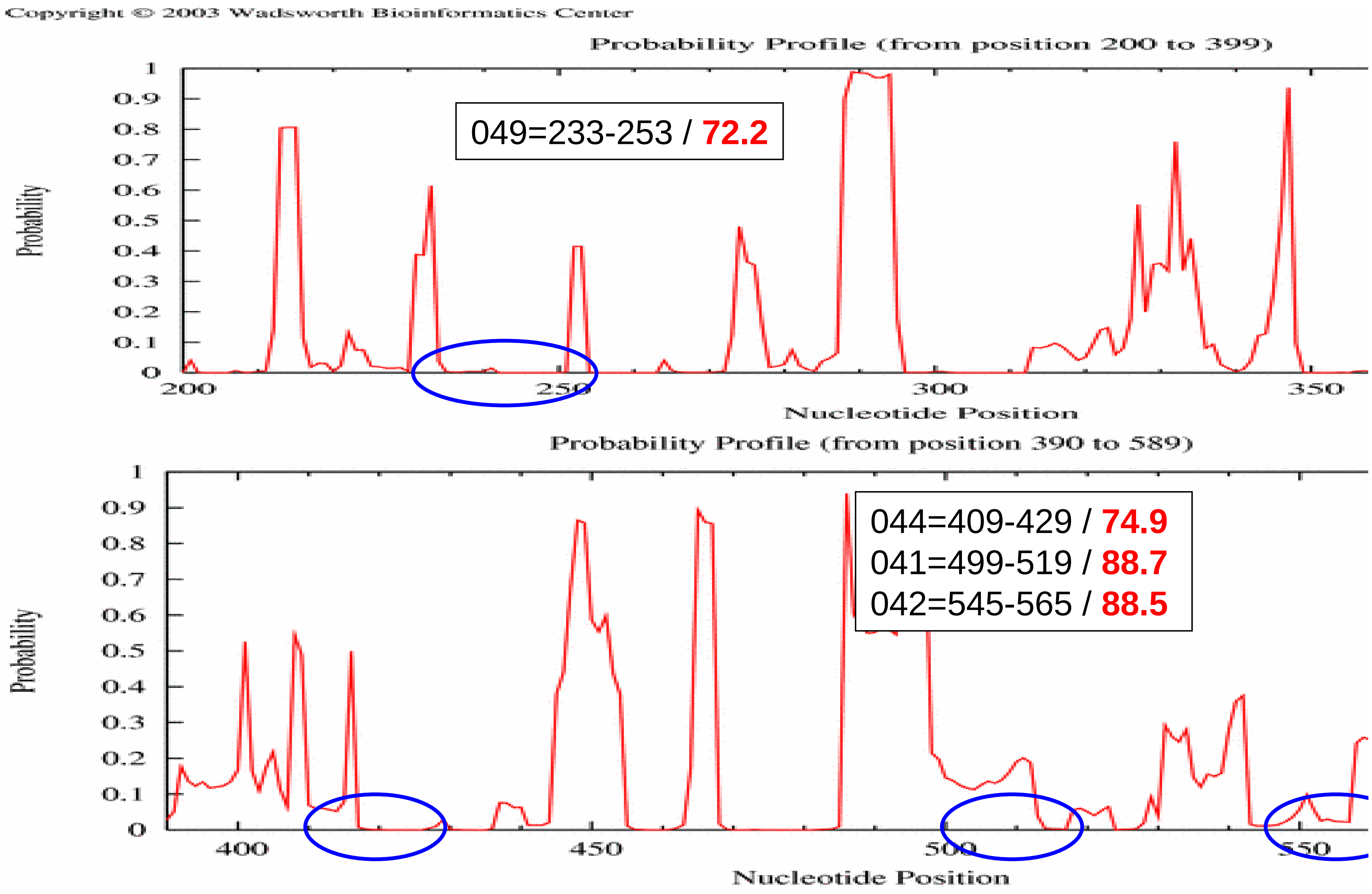

SFOLD probability profiling for target accessibility prediction : CSNK2A2

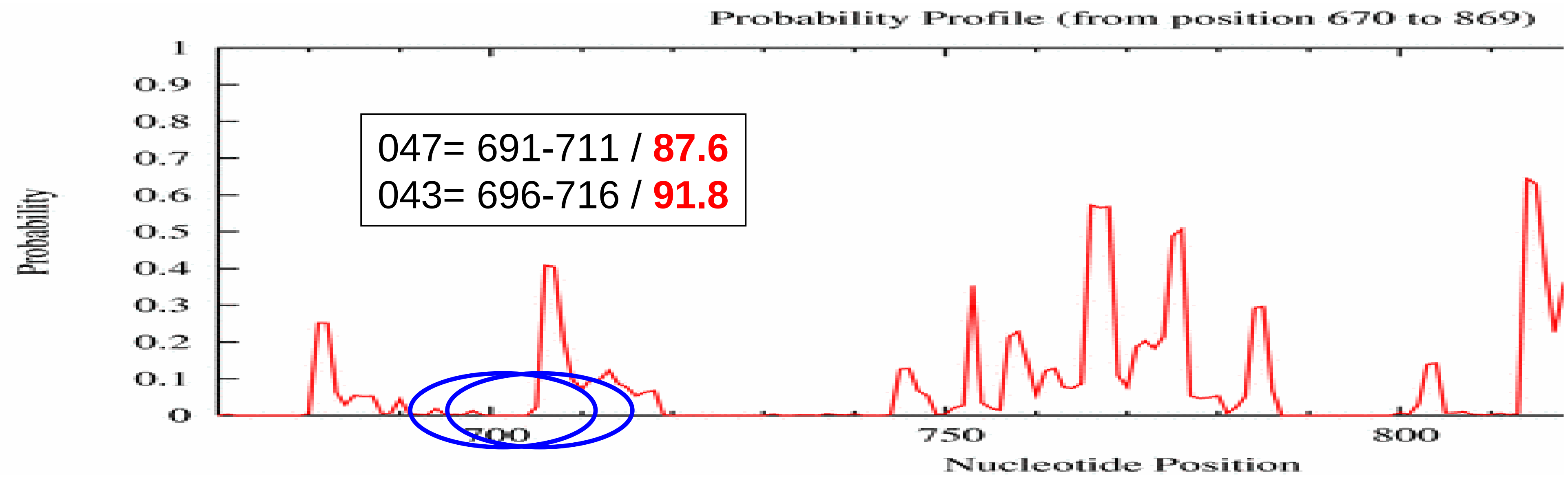

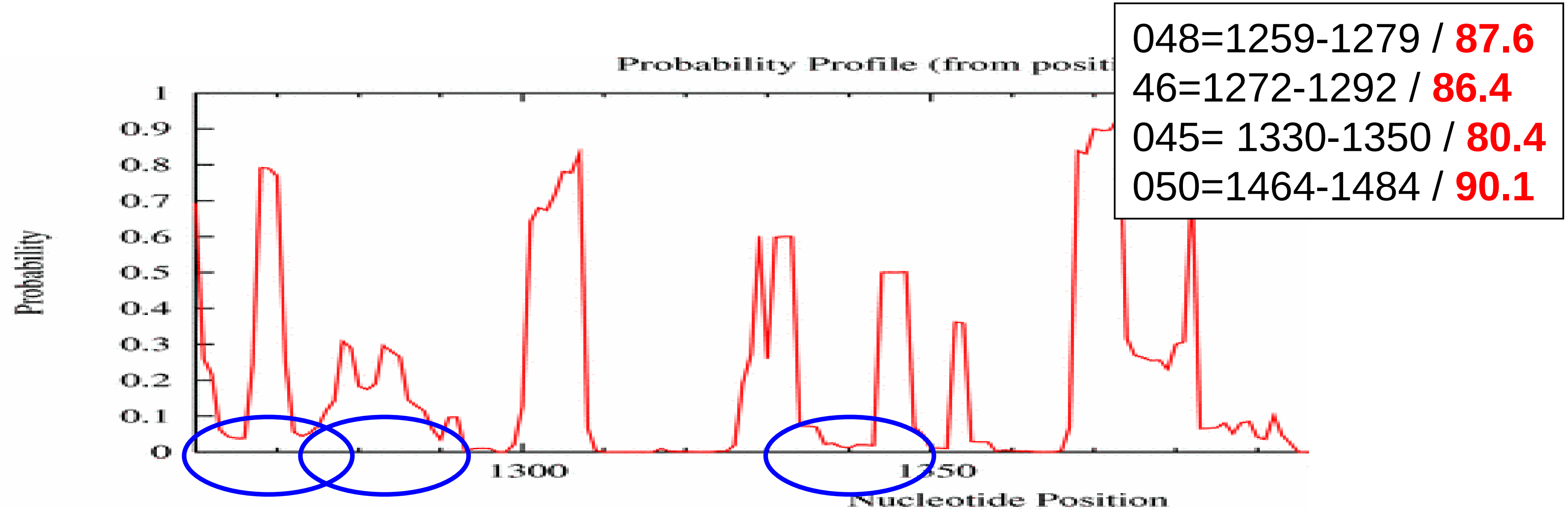

SFOLD probability profiling for target accessibility prediction : CSNK2B

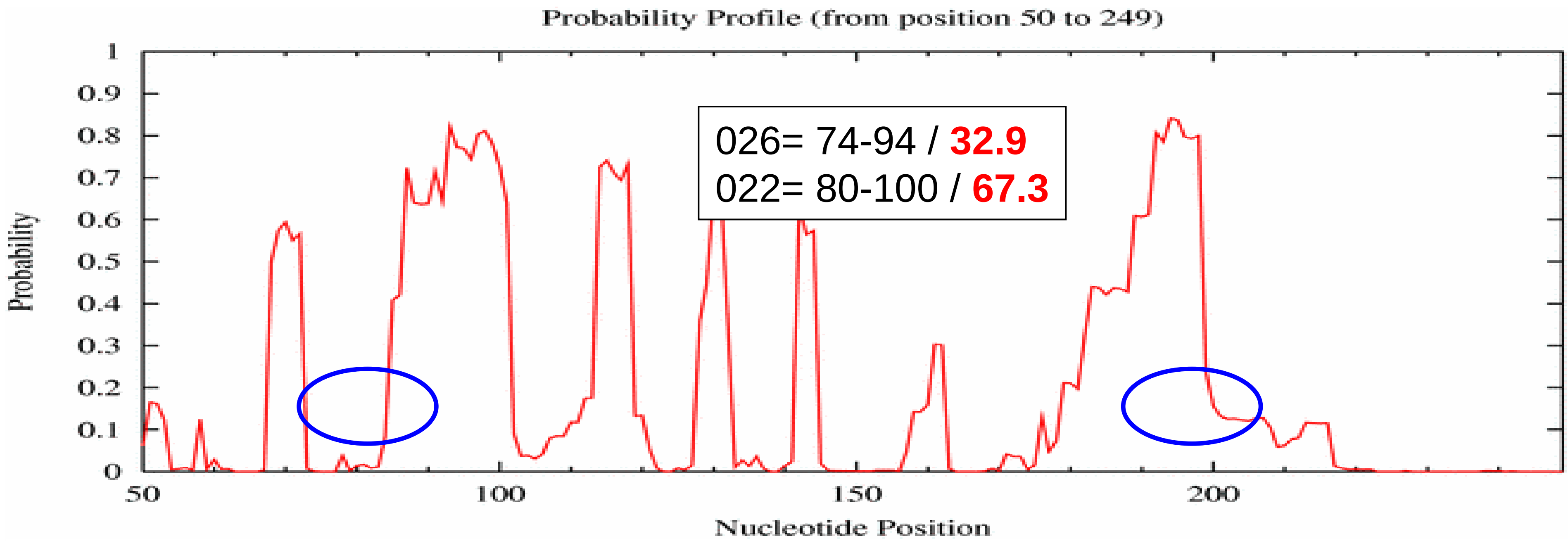

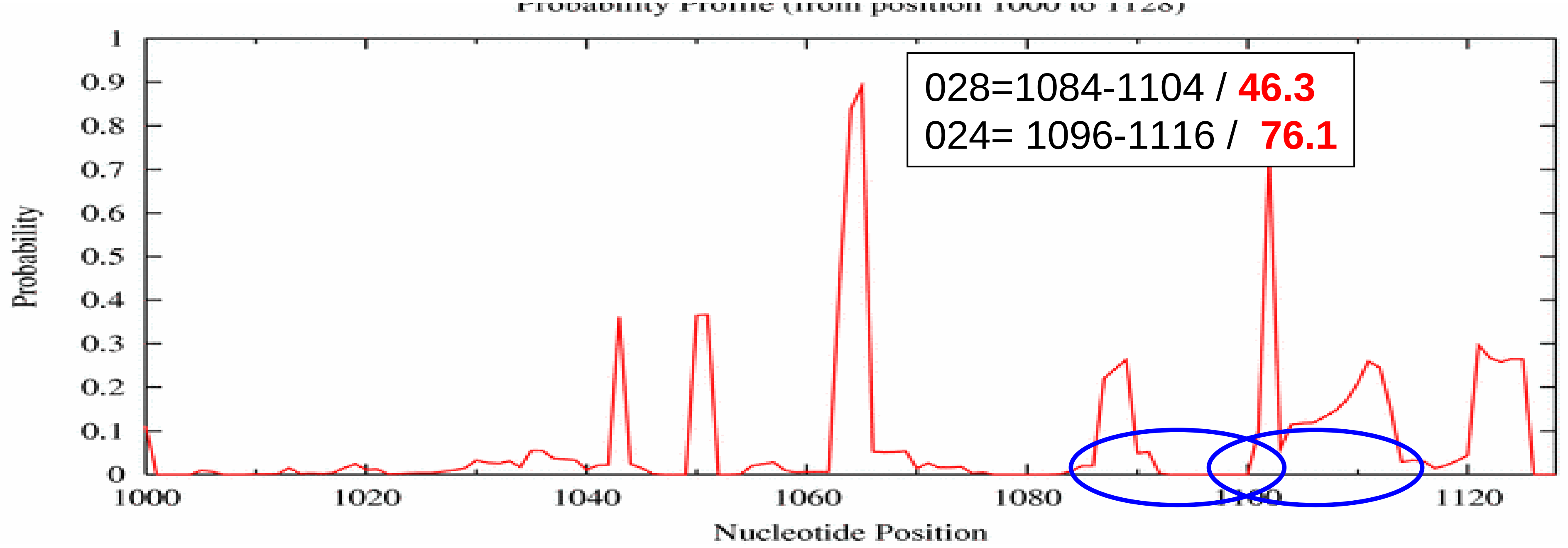

SFOLD probability profiling for target accessibility prediction : CSNK2B

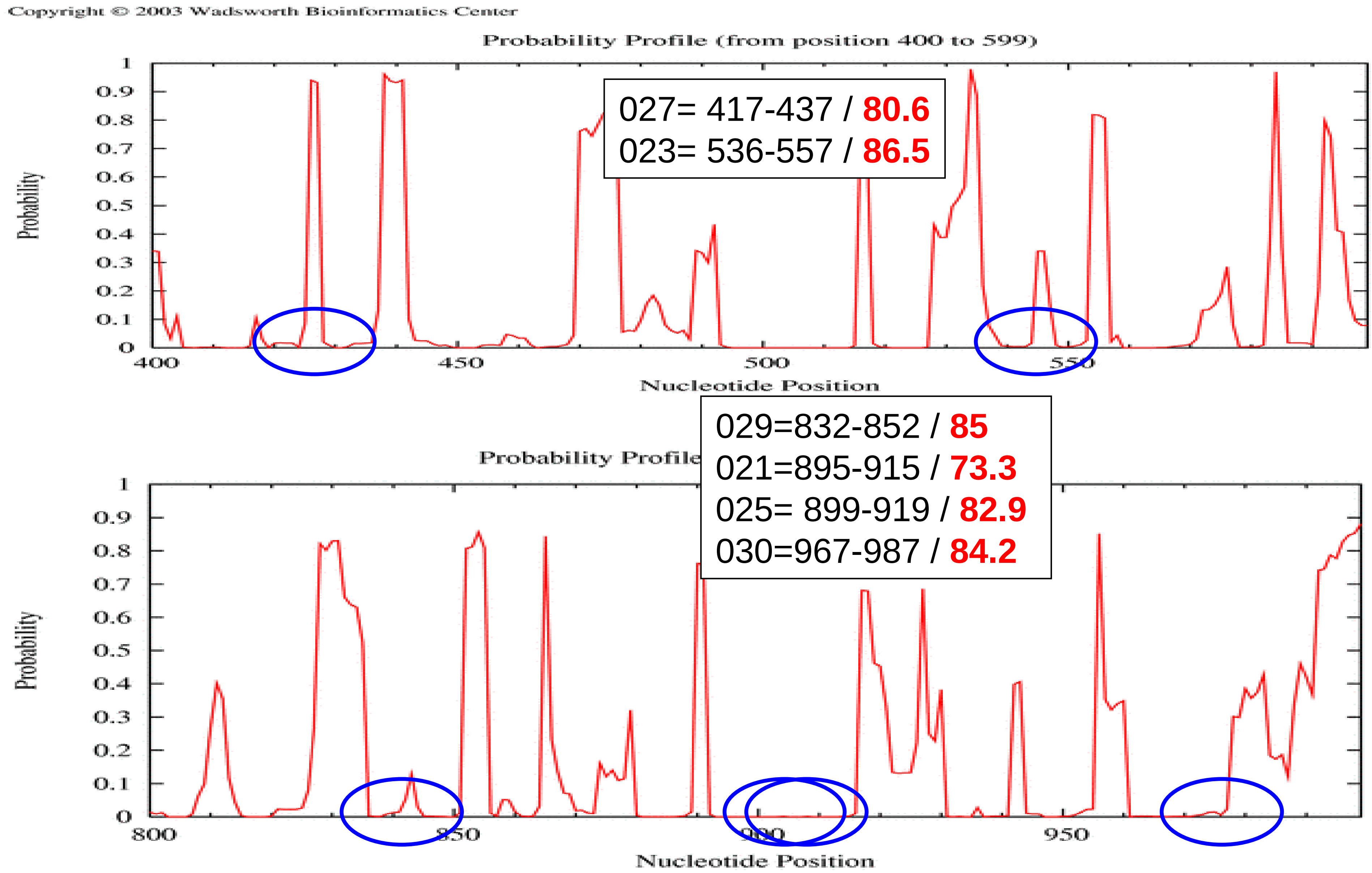

SFOLD probability profiling for target accessibility prediction : CSNK2A1

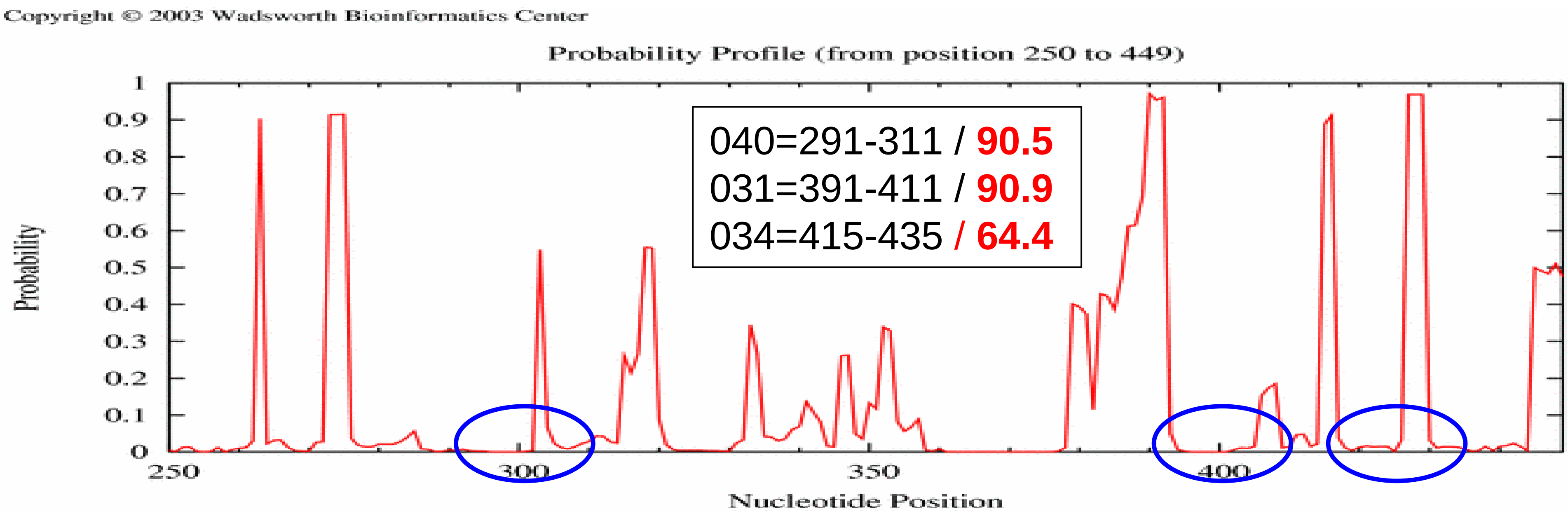

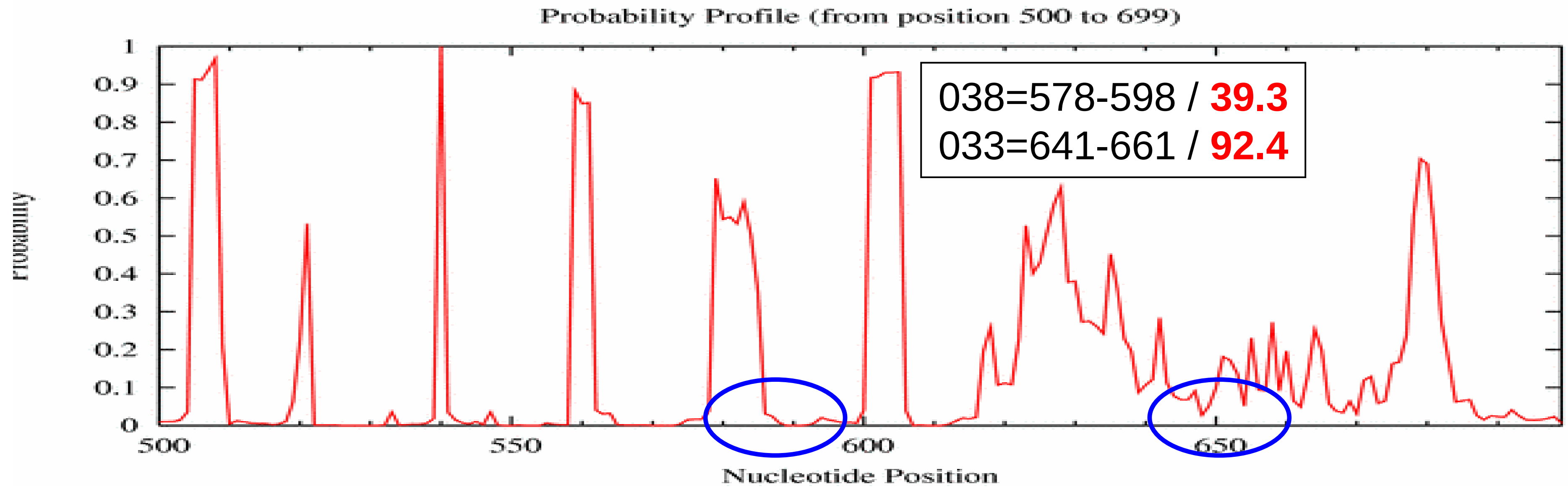

SFOLD probability profiling for target accessibility prediction : CSNK2A1

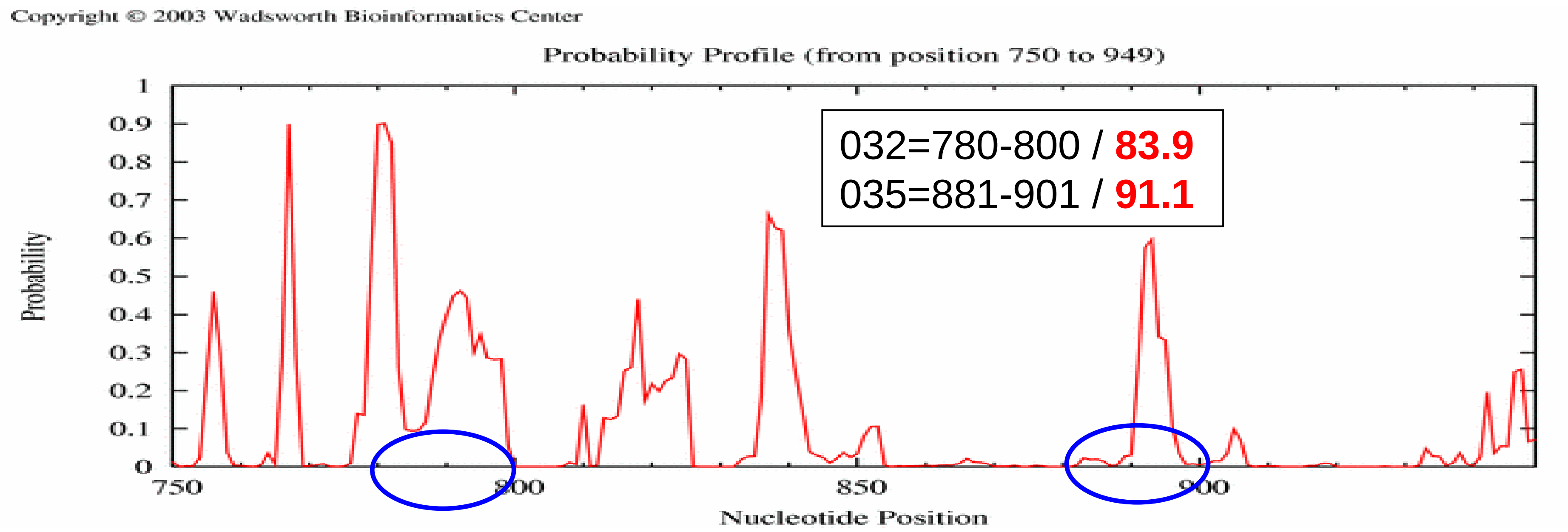

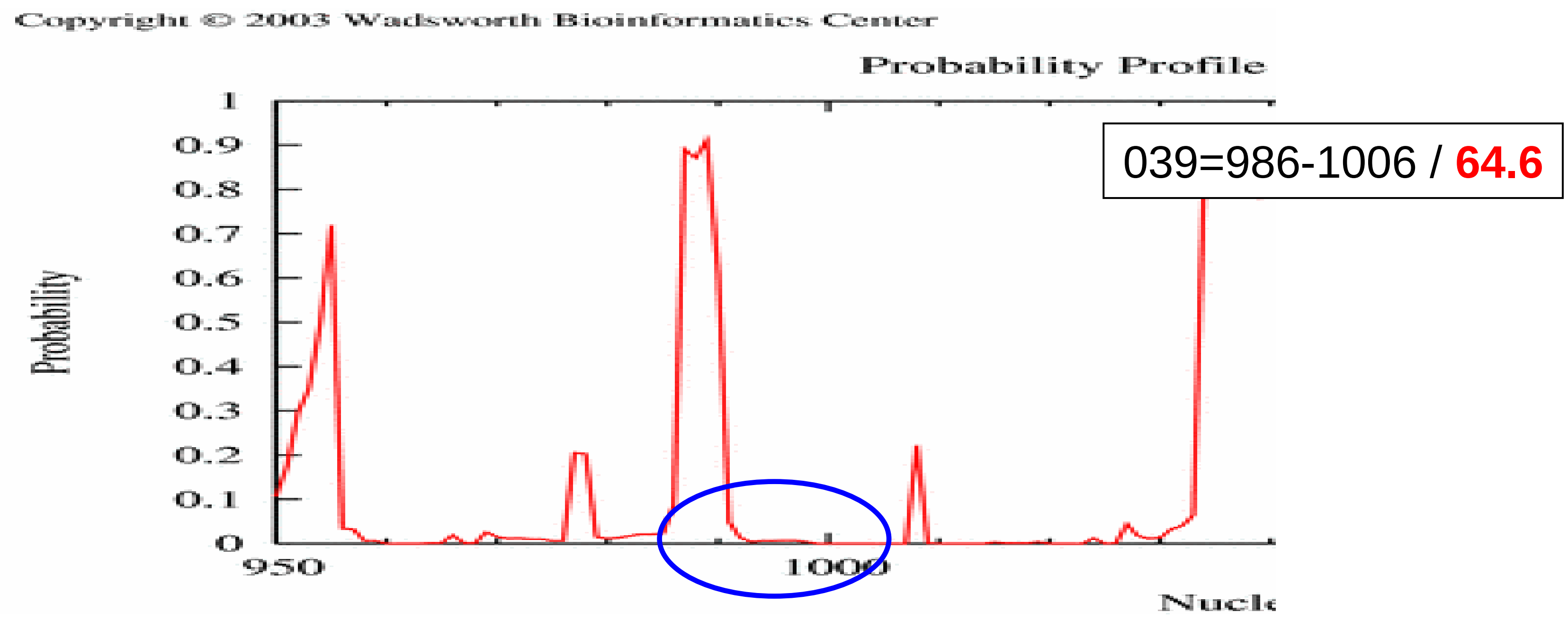

SFOLD probability profiling for target accessibility prediction : CSNK2A1

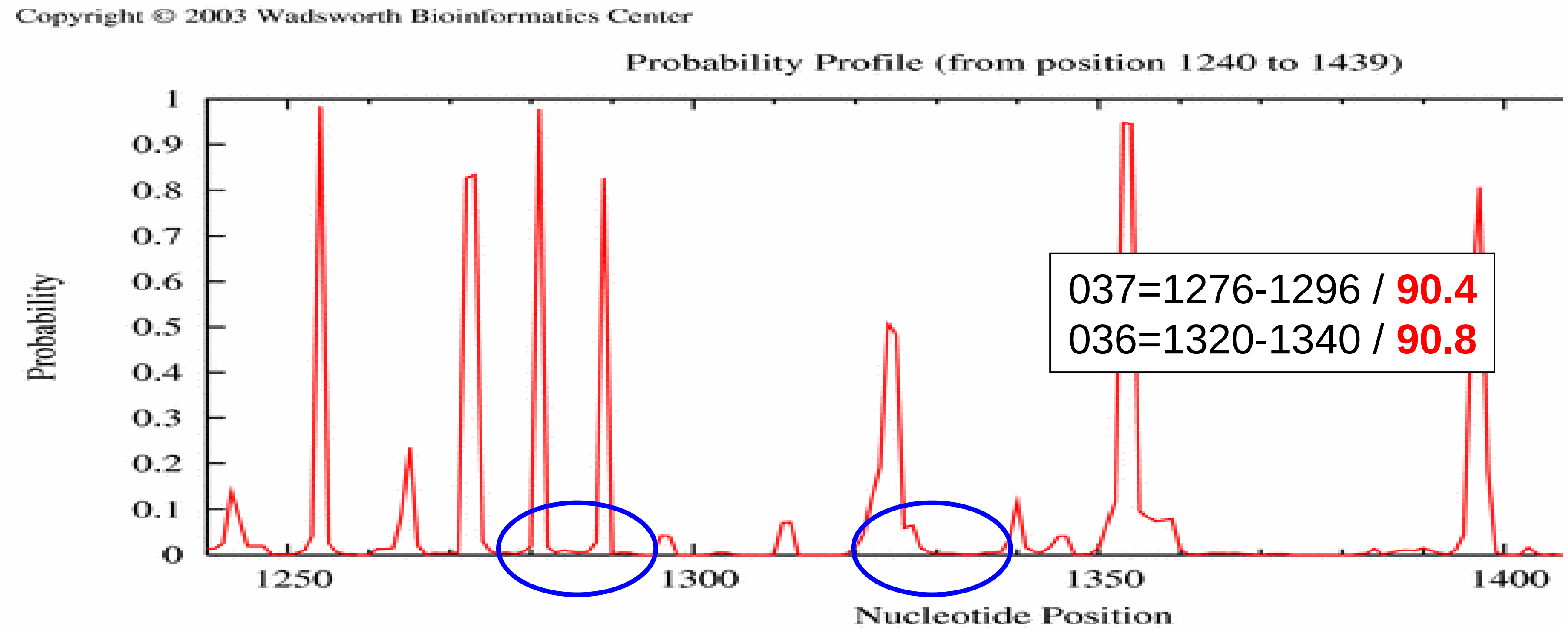

SFOLD probability profiling for target accessibility prediction : HIF1A

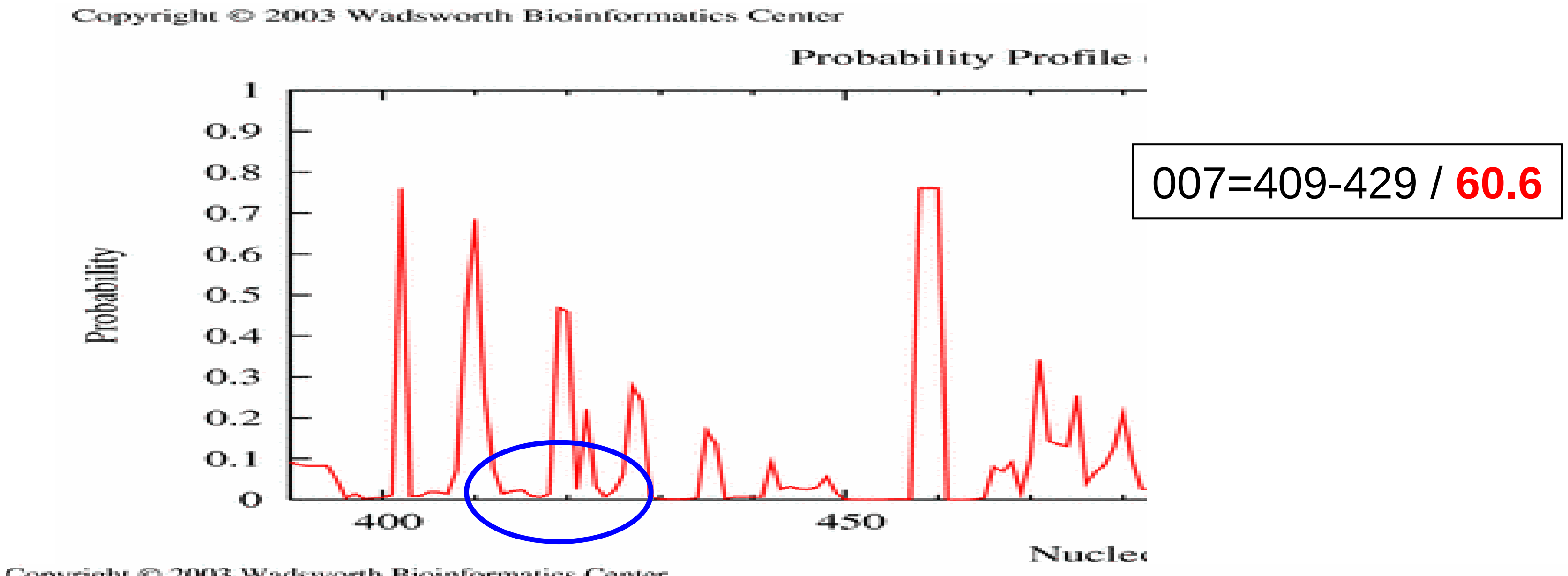

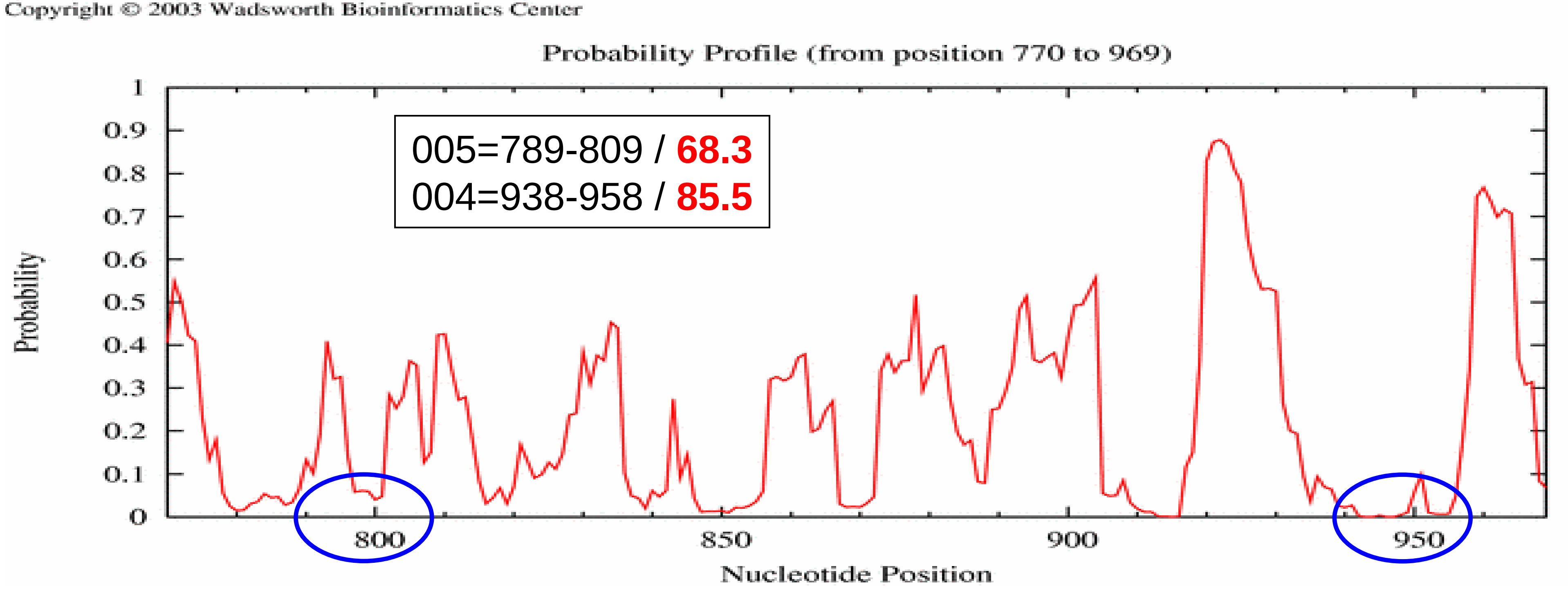

SFOLD probability profiling for target accessibility prediction : HIF1A

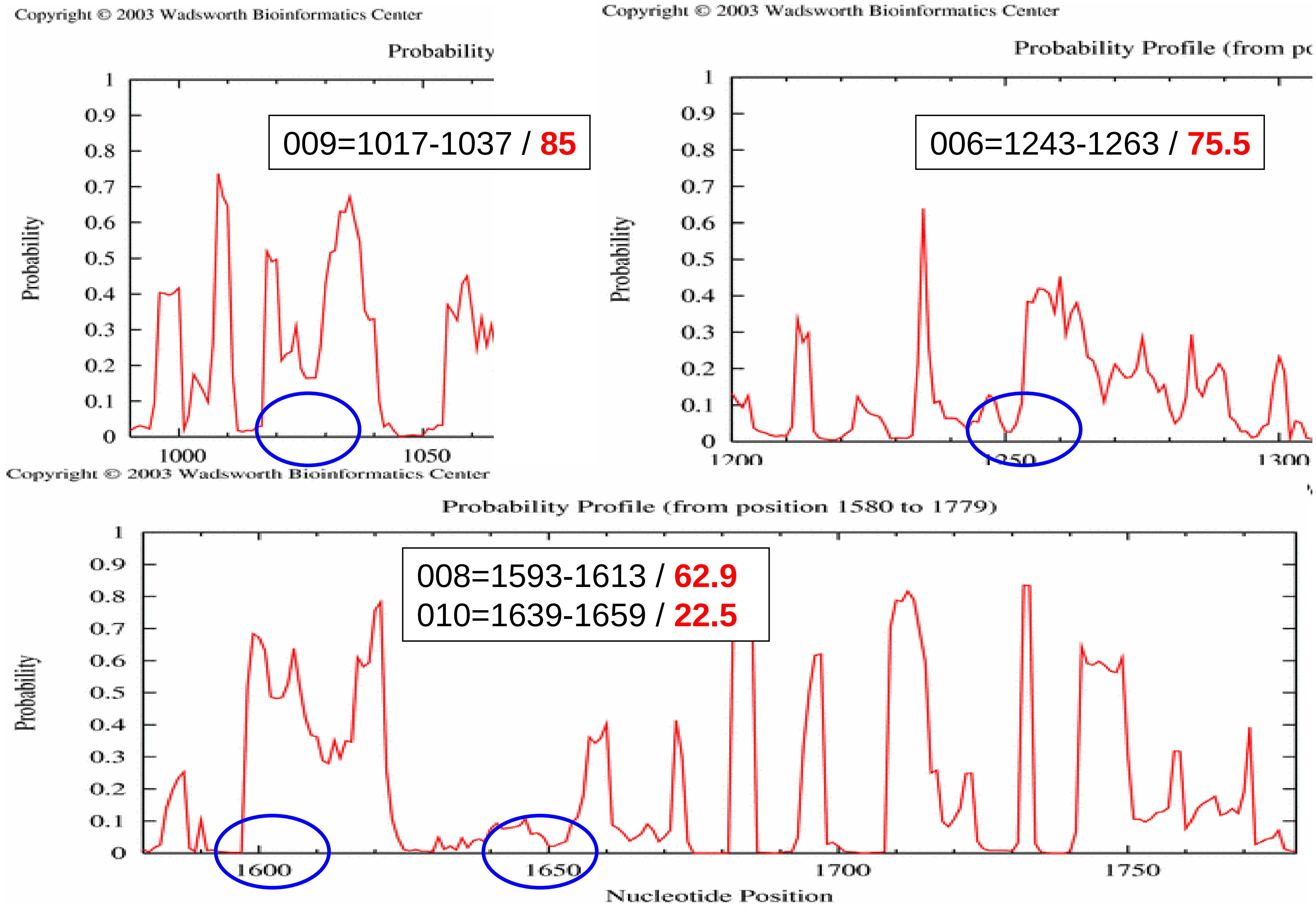

SFOLD probability profiling for target accessibility prediction : HIF1A

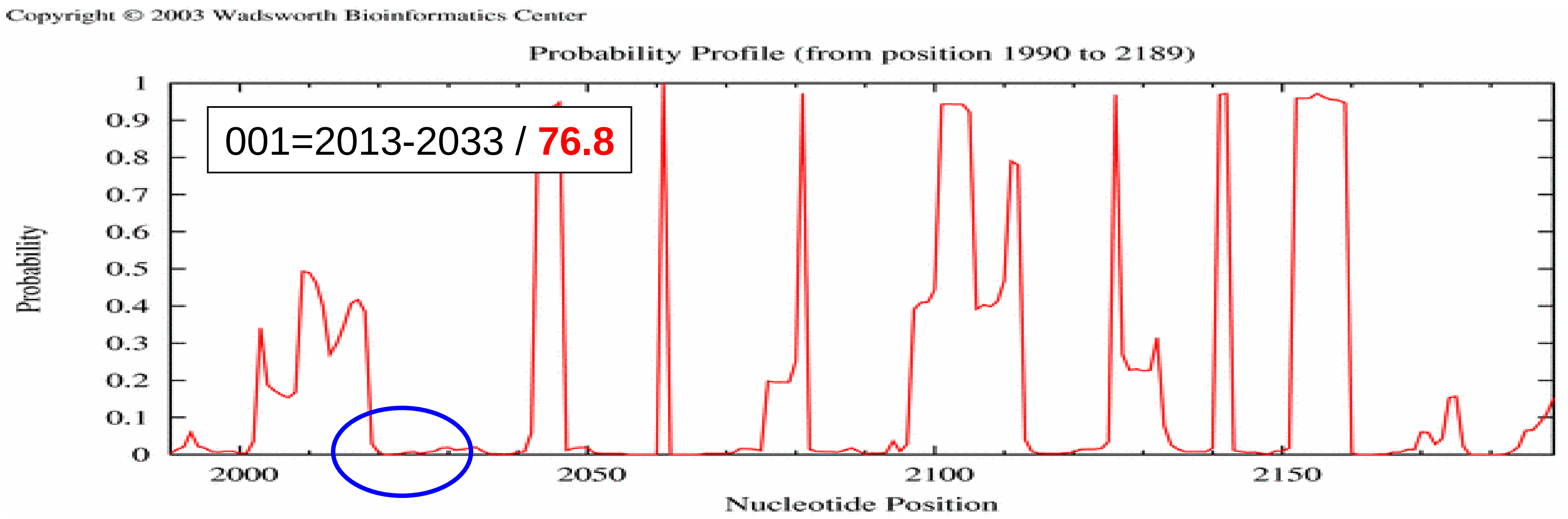

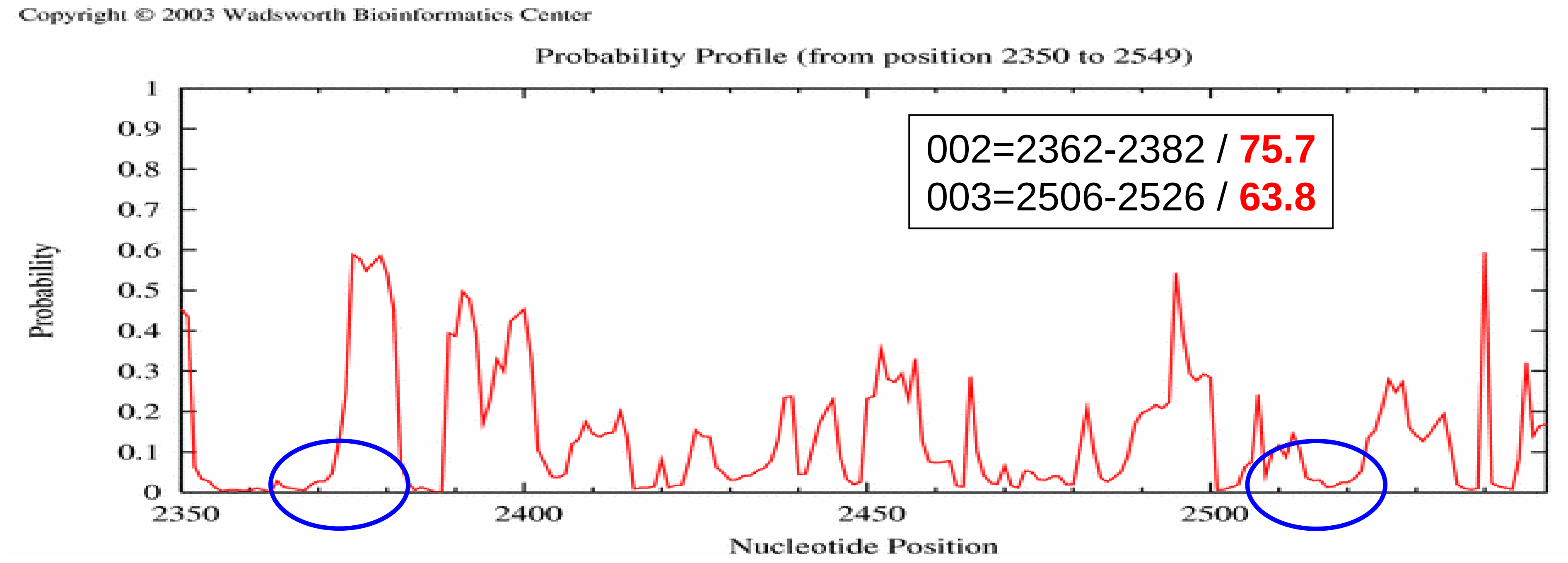

SFOLD probability profiling for target accessibility prediction : ERCC1

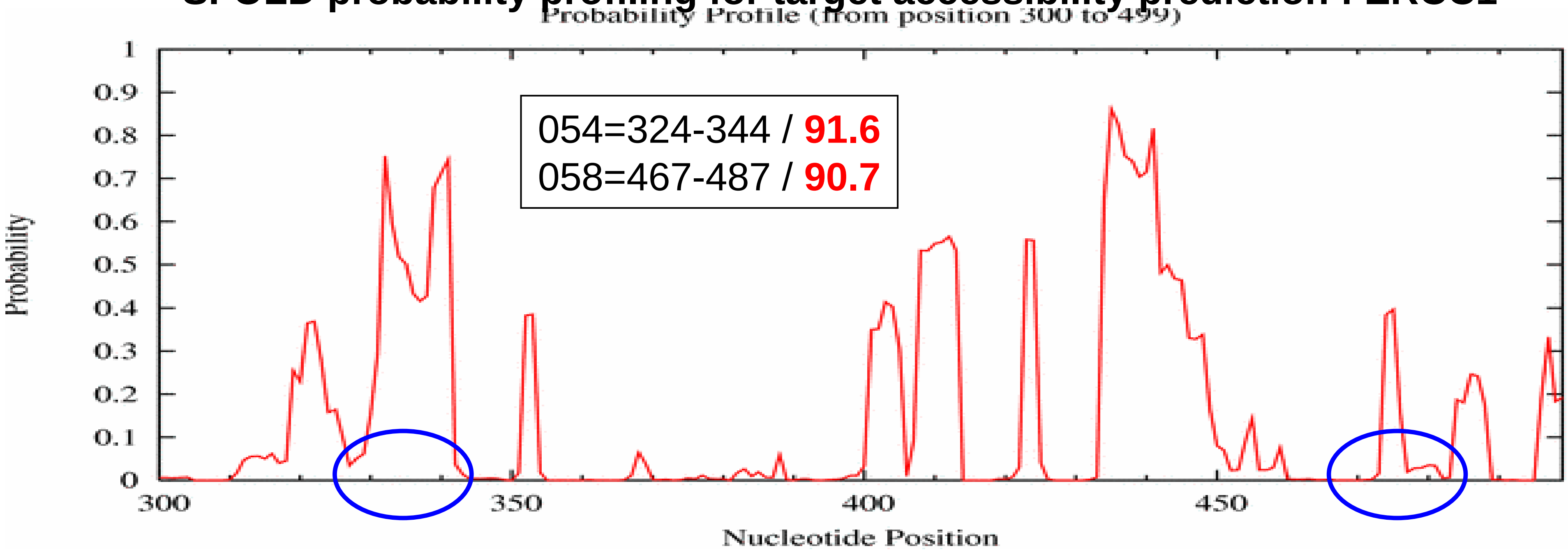

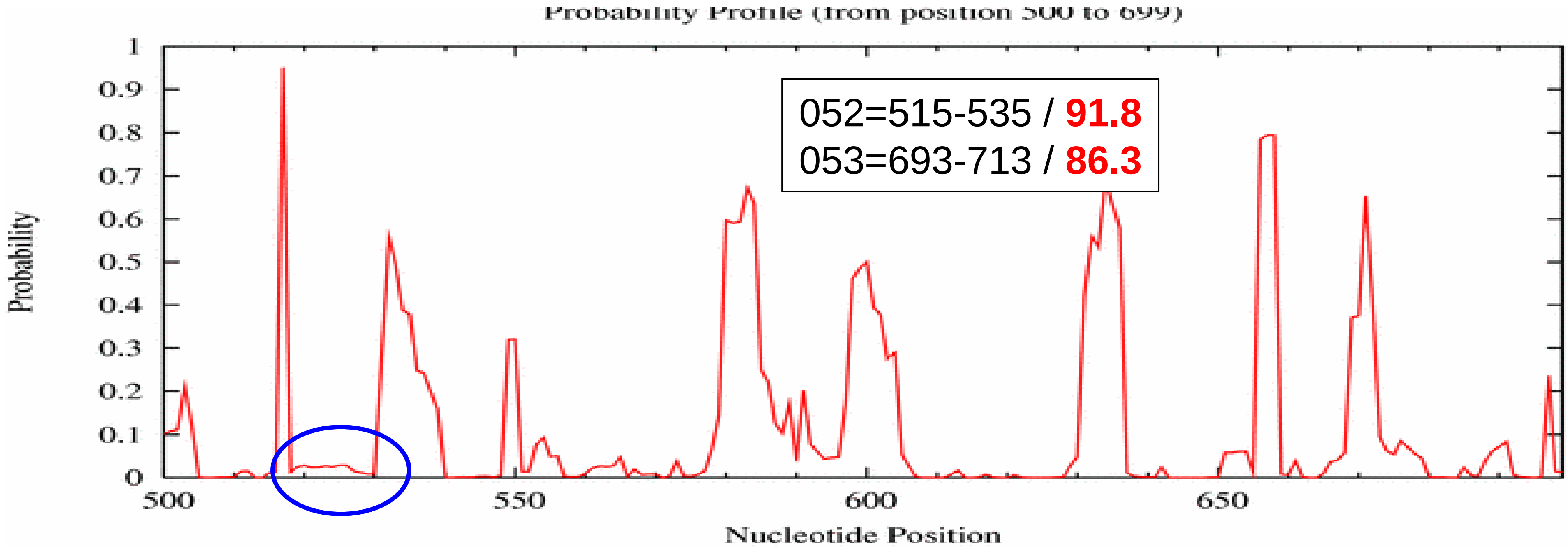

SFOLD probability profiling for target accessibility prediction : ERCC1

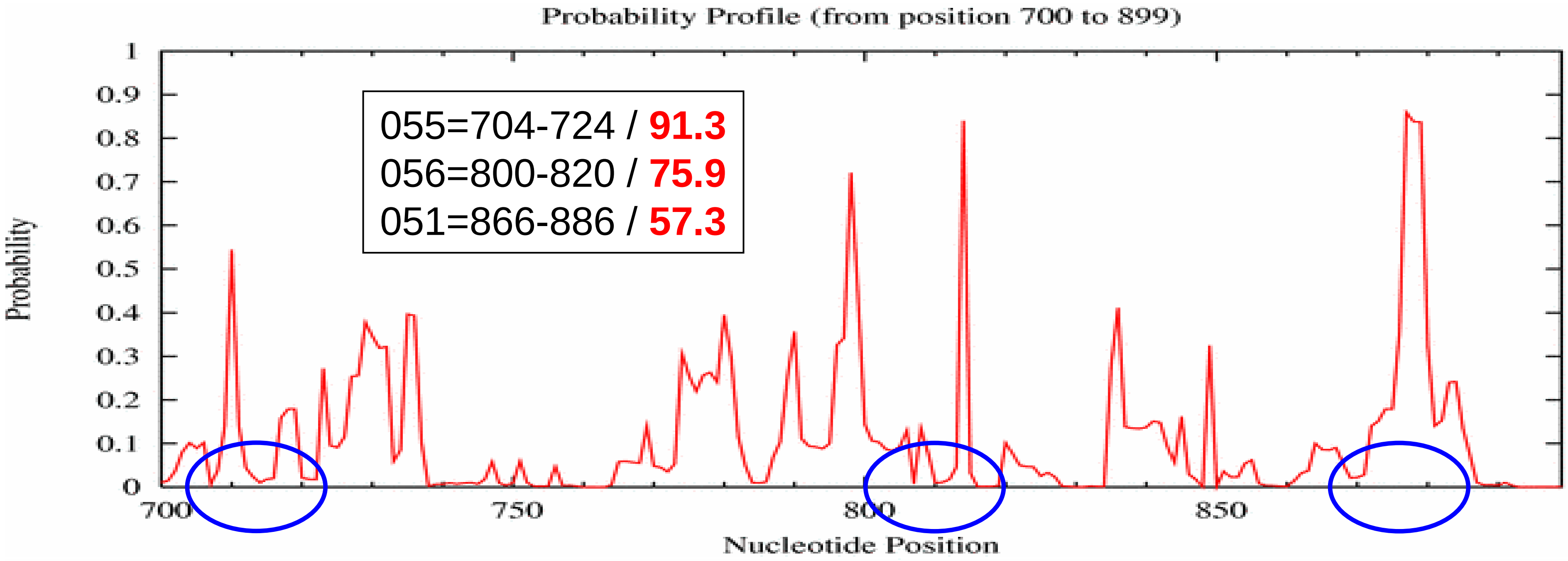

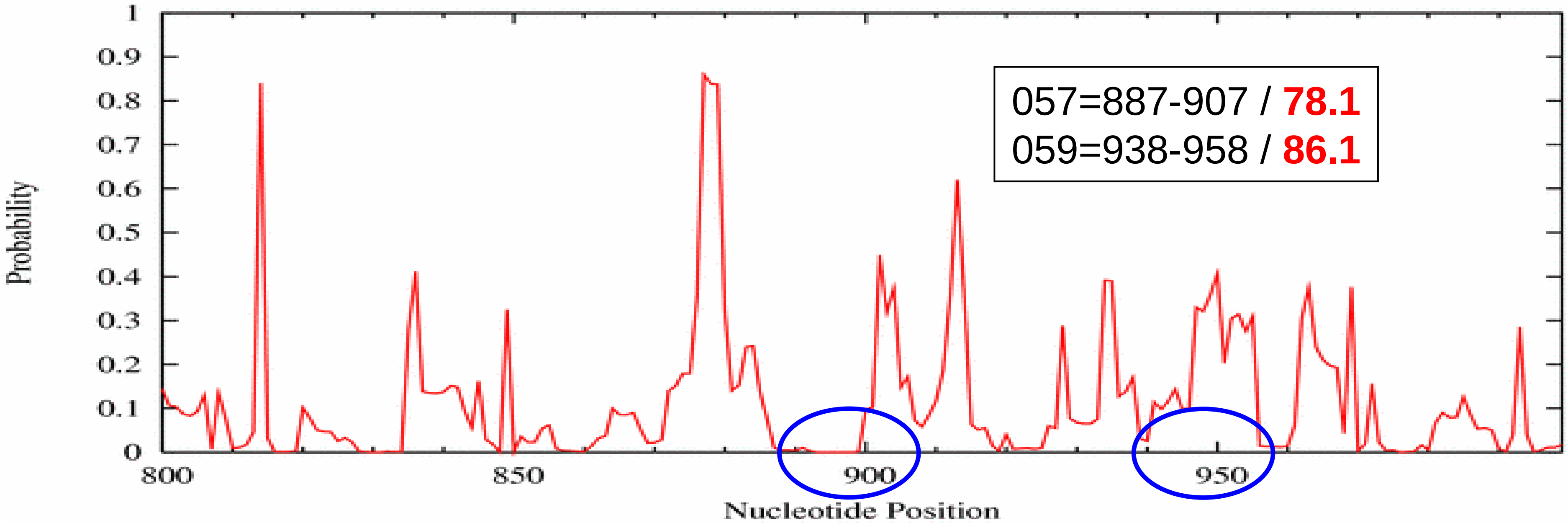

Supplement: Figure S2 — Target accessibility prediction profile for the eight mRNA targets and 88 corresponding siRNA sequences. Each full-length sequence target was submitted to the SFold server (siRNA section - http://sfold.wadsworth.org/cgi-bin/sirna.pl). The target accessibility probability profile for each site targeted by the siRNA is displayed. Blue circle highlights target sites for a given siRNA guide strand. For each siRNA, information in the box indicates: its identifier, start and end positions in the target and the knockdown activity measured (in bold red). (PDF) [file pone.0048057.s002.pdf]
