# Supplementary material for: DSIR: Assessing the Design of Highly Potent siRNA by Testing a Set of Cancer-Relevant Target Genes
Source: PLoS One. 2012 Oct 30;7(10):e48057. doi: 10.1371/journal.pone.0048057 (PMC3484153; doi:10.1371/journal.pone.0048057)
Supplement: Materials S1 — Materials and References. (DOC) [file pone.0048057.s006.doc]

**Materials S1**

**qRT-PCR data normalization and statistical analysis**

In this section, we present the statistical model used in our analysis. For general references on statistical analysis of PCR data see [1, 2, 3 and 4]. The analysis performed combined a linear model allowing the sources of variations in the experiment to be identified, and amplification efficiency to be estimated at various cycles based on a model. The procedure was as follows:

Let $E_{n}^{i}$ denote the amplification rate for molecule $i$ at cycle *n*. The concentration of this molecule, $Z_{n}^{i}$, is updated at each cycle according to:

Current concentration thus depends on the initial concentration through:

$$\log Z_{n}^{i}=\log Z_{0}^{i}+\sum_{k=0}^{n-1} \log\left(1+E_{k}^{i}\right)$$

Two series for each molecule during each PCR run were compared based on the number of cycles necessary to reach a given fluorescence threshold. Let and be these numbers for the control and treatment conditions (in practice, interpolation is required to determine these values, which are not integers). Assuming reproducibility, the observed is exactly the number of cycles required to compensate for the initial difference in concentration:

$$\log Z_{0}^{i}(tr)-\log Z_{0}^{i}(cnt)=\left(C^{i}(cnt)-C^{i}(tr)\right) \log\left(1+E_{0}^{i}\right)$$

Here we are assuming that amplification during the earlier stages of the process, when compensation takes place, is constant for the molecule considered. Normalization is required because the treatment and control are derived from two different biological samples. House-keeping genes were used for this purpose (referred to by a superscript $n$), giving estimates $Q^{*}$of the extinction rate $Q$:

$$Q=\frac{Z_{0}^{i}(tr)}{Z_{0}^{i}(cnt)}\frac{Z_{0}^{n}(cnt)}{Z_{0}^{n}(tr)}$$

$$Q^{*}=\frac{\left(1+E_{0}^{i}\right)^{C^{i}(cnt)-C^{i}(tr)}}{\left(1+E_{0}^{n}\right)^{C^{n}(cnt)-C^{n}(tr)}}$$

In our experiments, using three replicates, and two normalizing genes, 18 extinction ratio estimates were obtained for each target gene. These cannot be assumed to be independent, and a global analysis was performed, based on a linear model on the log scale.

This model is as follows, where *r* is the plate identifier and *u* is the treatment:

$$\log\left(1+E^{i}\right) C^{i}(u,r)=\alpha_{r}+\beta_{i}+\gamma_{u}+\delta_{ri}+\eta_{ru}+\tau_{iu}+\sigma\varepsilon_{riu}$$

Treatment contrasts were applied to each term apart from the plate term, . For instance, $\gamma_{u}\neq0$ only for the siRNA analyzed, and does not appear for the control condition. This term is the model predicting the increase in response resulting from targeting any molecule with the siRNA. The coefficient of interest is the interaction term, , which quantifies the effect of the treatment applied specifically to the target molecule.

To clarify this, consider the predicted response when only one plate is considered (in this case we can ignore the plate identifier, and the interaction terms $\delta_{ri}$ and $r_{ru}$ are absorbed into $\beta_{i}$ and $\gamma_{u}$, respectively). For the target gene, under treatment conditions, the predicted response is:

$$\log\left(1+E^{i}\right) C^{i}(u)=\alpha+\beta+\gamma+\tau$$

In contrast, for the same molecule, under control conditions, we have the following prediction:

$$\log\left(1+E^{i}\right) C^{i}(0)=\alpha+\beta+0+0$$

The respective corresponding predictions for the normalizing gene are:

$$\log\left(1+E^{0}\right) C^{0}(u)=\alpha+0+\gamma+0$$

and:

$$\log\left(1+E^{0}\right) C^{0}(0)=\alpha+0+0+0$$

The interaction term is thus:

$$\tau=\log\left(1+E^{i}\right) (C^{i}(u)-C^{i}(0))-\log\left(1+E^{0}\right) (\text{C}^{0}\text{(u)-C}^{0}(0))$$

This is precisely the logarithm of the estimate $Q^{*}$ in this simplified context where only one plate and one normalizing gene are considered.

Homogeneity and normality of the residuals were checked, confirming that the model is appropriate, assuming known initial amplifications$E^{i}$, confidence intervals for , and one-sided p-values were obtained.

The procedure outlined does not take $E^{i}$ uncertainty into account. It is widely acknowledged that this efficiency varies rapidly during amplification cycles [6,7], and could thus affect concentration estimates. Our approach to this problem was to conduct two separate analyses, each based on a different method. The first makes use of dilution series, acquired at a calibration stage; the second is based on a statistical model of the apparent amplifications as a function of the cycle number. Extrapolation of this model to the origin (at which point the signal does not yet emerge from noise, making a direct estimation impossible) provides us with the required values. These two procedures produced only minor differences. Thus, the conclusions remain the same.

**References**

1 - Pfaffl MW: A new mathematical model for relative quantification in real-time RT-PCR. Nucl Acids Res 2001, 29:2002-2007.

2 - Yuan JS, Reed A, Chen F, Stewart CN: Statistical analysis of real-time PCR data

BMC Bioinformatics 2006, 7:85.

3 - Gibson UE, Heid CA, Williams PM: A novel method for real time quantitative RT-PCR. Genome Res 1996, 6:995-1001.

4 - Nolan, T., Hands, R. E., & Bustin, S. A. (2006). Quantification of mRNA using real-time PCR. Nat Protoc, 1, 1559-1582.

5 - Larionov A, Krause A, Miller W: A standard curve based method for relative real time PCR data processing. BMC Bioinformatics 2005, 6:62.

6 - Yuan, J. S., Wang, D., & Stewart, C. N. (2008). Statistical methods for efficiency adjusted real-time PCR quantification. Biotechnology Journal, 3(1), 112-123.

7 - Platts, A. E., Johnson, G. D., Linnemann, A. K., & Krawetz, S. A. (2008). Real-time PCR quantification using a variable reaction efficiency model. Analytical Biochemistry, 380(2), 315-322.
